# The Effects of Seaweed and Microalgae Supplementation on Exercise Performance and Recovery: A Systematic Review and Meta-Analysis

**DOI:** 10.3390/nu18081289

**Published:** 2026-04-19

**Authors:** Yan Wei, Shuning Liu, Ting You, Xingyu Liu, Wen Zhong, Yutong Wu, Samuhaer Azhati, Qisen Han, Wei Jiang, Chang Liu

**Affiliations:** 1 School of Education, Beijing Sport University, Beijing 100084, China; 2024011355@bsu.edu.cn (Y.W.); sam@bsu.edu.cn (S.A.); 2 School of Sports Science, Beijing Sport University, Beijing 100084, China; 2023013553@bsu.edu.cn (S.L.); 2025012597@bsu.edu.cn (W.Z.); 2024012786@bsu.edu.cn (Y.W.); work@bsu.edu.cn (Q.H.); 3 School of Leisure Sports and Tourism, Beijing Sport University, Beijing 100084, China; t.you@bsu.edu.cn; 4 School of Basic Medical Sciences, Peking University Health Science Center, Beijing 100191, China; 2310122714@stu.pku.edu.cn; 5 China Volleyball Academy, Beijing Sport University, Beijing 100084, China

**Keywords:** seaweed, spirulina, chlorella, sports nutrition, exercise performance, muscle damage, meta-analysis

## Abstract

**Objective:** Seaweed and microalgae provide antioxidants, polyunsaturated fatty acids, and bioactive compounds that may enhance exercise performance and accelerate recovery. However, evidence remains inconsistent. This systematic review and meta-analysis aimed to evaluate the effects of algae-derived supplementation on exercise performance and physiological recovery outcomes in healthy and athletic adults. **Methods:** This review was registered in PROSPERO (CRD420251166723) and conducted in accordance with PRISMA 2020 guidelines. PubMed, Web of Science, Embase, Cochrane Library, EBSCO, and CNKI were systematically searched for randomized controlled trials (RCTs) evaluating algae supplementation in exercise contexts. Inclusion and exclusion criteria were defined based on the PICOS framework. Primary outcomes included VO_2_max, Time to exhaustion (TTE), maximal power output (WRmax), Time-Trial (TT) performance, and creatine kinase (CK). Standardized mean differences (SMDs) with 95% confidence intervals (CIs) were calculated using a random-effects model. Subgroup, sensitivity, and publication bias analyses were performed. **Results:** Twenty-two RCTs (*n* = 822) investigating Spirulina, Chlorella, brown-algal polysaccharides, or astaxanthin met inclusion criteria. Algae supplementation showed a suggestive improvement in VO_2_max (SMD = 0.88, 95% CI: 0.00–1.75) and significantly improved in TTE (SMD = 1.06, 95% CI: 0.16–1.96), with smaller effects on WRmax (SMD = 0.29, 95% CI: 0.03–0.55), and no significant benefit for TT performance (SMD = −0.27, 95% CI: −0.74 to 0.21). Regarding recovery, CK concentrations were significantly reduced (SMD = −0.78, 95% CI: −1.28 to −0.28). Subgroup analysis suggested greater effects for Chlorella supplementation, higher dosages, and aerobic training contexts; reductions in muscle-damage markers were more evident following resistance exercise. Sensitivity analyses supported the robustness of the main findings with minimal evidence of publication bias. **Conclusions:** Algae-derived supplements—particularly Spirulina and Chlorella—may modestly enhance aerobic exercise performance and attenuate exercise-induced muscle damage under certain conditions. Effects appear to depend on algae species, dosing strategies, intervention duration, and training modality. High-quality, multi-center RCTs incorporating mechanistic endpoints are needed to clarify optimal application and to develop athlete-specific recommendations.

## 1. Introduction

Seaweeds (macroalgae, including brown, red, and green algae) and microalgae (e.g., Spirulina, Chlorella, *Haematococcus pluvialis*) are rich sources of high-quality protein, essential amino acids, B vitamins, and trace elements such as iron and magnesium [1]. They also contain diverse natural bioactive compounds, including phycocyanin, carotenoids (e.g., β-carotene, lutein, astaxanthin), polyphenols, and sulfated polysaccharides [2,3]. This unique nutritional profile confers high nutrient density and provides a mechanistic basis for their potential roles in modulating energy metabolism, redox homeostasis, and inflammatory responses—processes that are closely linked to exercise performance and recovery [4].

Mechanistically, seaweed- and microalgae-derived compounds may influence exercise capacity and post-exercise recovery through several interrelated pathways. First, with respect to oxygen transport and hematological adaptations, certain algae are abundant in iron, chlorophyll, and related co-factors [5], which may support erythropoiesis and increase hemoglobin concentration. These adaptations could enhance oxygen delivery and utilization, potentially contributing to improvements in maximal oxygen uptake (VO_2_max) [6]. Second, in relation to vascular function and mitochondrial metabolism, algae-derived nitrate precursors, arginine, and polyphenols may enhance endothelial function and nitric oxide bioavailability, thereby improving muscle perfusion and oxygen efficiency during exercise. Such effects may optimize aerobic energy production and delay the onset of metabolic fatigue [7]. Third, in terms of antioxidant, anti-inflammatory, and immunomodulatory actions, carotenoids, polyphenols, and sulfated polysaccharides have been shown to scavenge reactive oxygen species, stabilize cellular membrane structures, and downregulate pro-inflammatory signaling pathways such as NF-κB [8,9,10]. These mechanisms may attenuate exercise-induced oxidative stress and muscle damage, reduce secondary inflammatory responses, and facilitate recovery processes.

Over the past two decades, multiple randomized controlled trials (RCTs) have investigated the effects of algae-derived supplementation in healthy and athletic populations [11,12,13]. Whole-algae preparations, such as *Spirulina* and *Chlorella*, have been reported in some studies to improve VO_2_max, prolong time to exhaustion (TTE), and reduce post-exercise lactate and creatine kinase (CK) concentrations [14]. Brown algal extracts, including polysaccharides and polyphenols, have demonstrated anti-fatigue and immunomodulatory properties in animal models and early-phase human studies [15]. Astaxanthin derived from *Haematococcus pluvialis* has attracted considerable attention due to its potent antioxidant capacity; however, evidence regarding its effects on exercise performance and recovery remains inconsistent [16,17]. Collectively, these findings suggest that seaweed and microalgae supplementation may enhance endurance-related performance and attenuate exercise-induced muscle damage. Nevertheless, interpretation is limited by small sample sizes, heterogeneity in supplementation protocols (e.g., species, dosage, duration, and formulation), variability in participant training status, and inconsistent outcome measures. These methodological differences contribute to uncertainty regarding the magnitude, direction, and stability of observed effects.

In light of these gaps, the present study conducted a systematic review and meta-analysis of RCTs examining oral supplementation with seaweed or microalgae in healthy adults and athletic populations. The primary aim was to quantitatively synthesize the effects of different algae species and preparations on exercise performance and post-exercise recovery outcomes. Predefined primary outcomes included VO_2_max, TTE, maximal workload or power output (WRmax), and CK concentrations. Secondary outcomes encompassed lactate dehydrogenase (LDH), markers of inflammation and oxidative stress, and subjective recovery indices. Subgroup analyses were conducted according to exercise modality, algae species, dosage, intervention duration, and preparation form. By integrating available evidence, this study sought to evaluate the dual effects of algae supplementation on exercise performance and recovery, identify intervention characteristics associated with greater efficacy, and provide more targeted evidence for sports nutrition practice, and guide the design of future high-quality RCTs.

## 2. Methods

### 2.1. Study Protocol Registration

The protocol for this systematic review and meta-analysis was pre-registered on the PROSPERO (International Prospective Register of Systematic Reviews) platform (Registration Number: CRD420251166723). It predefined the research background, PICOS framework, primary and secondary outcomes, literature search strategy, and statistical analysis plan, following PRISMA 2020 and Cochrane Handbook requirements [18,19]. To ensure transparency, we note that, prior to data synthesis, we expanded the list of exploratory outcomes to include metabolic and oxidative stress markers, and adjusted the database search dates to ensure comprehensive coverage of the most recent clinical evidence through June 2025. Additionally, some pre-specified subgroup analyses could not be fully conducted due to insufficient study data in certain categories. These modifications did not alter the primary research questions or core outcomes (e.g., VO_2_max, TTE, WRmax, CK).

### 2.2. Literature Search Strategy

This study followed the PRISMA 2020 reporting guidelines [18,20]. The search and screening process was designed based on the PICOS principle (Population, Intervention, Comparator, Outcomes, Study design). The search framework and keyword strategy were developed with reference to our previously published systematic review in the field of exercise-related nutritional supplementation [21,22].

Two researchers independently conducted the systematic search, with the search cutoff date being June 2025. Databases searched included PubMed, Web of Science, Embase, EBSCO, Cochrane Library, and CNKI (China National Knowledge Infrastructure). These databases were selected to provide optimal indexing coverage across the fields of exercise physiology, sports nutrition, and pharmacological research. To capture the broadest relevant evidence, the search strategy combined both subject headings and free-text terms. Intervention-related keywords included “Spirulina,” “Arthrospira,” “Chlorella,” “Seaweed,” “Microalgae,” “Phytoplankton,” “Brown algae,” “Ecklonia cava,” “Fucoidan,” “Fucoxanthin,” “Astaxanthin.” Exercise-related keywords included “exercise,” “physical performance,” “endurance,” “fatigue,” “recovery.” Boolean operators “AND” and “OR” were used for combination.

The search was conducted from database inception until June 2025, with no restriction on publication date. To ensure methodological rigor, we initially did not apply any language filters to our search strategy. Our systematic search across the selected databases yielded zero (*n* = 0) relevant records in languages other than English and Chinese during the initial identification and screening phases. While we acknowledge that our primary reliance on these specific databases may inherently limit the capture of trials published in other regional languages, previous methodological evidence [23] suggests that the exclusion of non-English trials typically exerts a minimal impact on the overall effect estimates in biomedical meta-analyses. Therefore, even if additional relevant studies existed in databases not covered by our search, they would be unlikely to substantively alter our core findings. Nevertheless, the final inclusion was limited to English and Chinese publications, and we acknowledge this as a methodological limitation.

Search results were imported into EndNote 2025 (Clarivate Analytics, Philadelphia, PA, USA) for deduplication. Two researchers (Y.W., S.L.) independently screened titles and abstracts, excluding records clearly irrelevant to the topic. Full texts of potentially eligible studies were retrieved and assessed for final inclusion. Disagreements were resolved through discussion or arbitration by a third researcher (C.L.). Authors were contacted for additional information when necessary.

### 2.3. Inclusion and Exclusion Criteria (PICOS)

Population: Included healthy adults (≥18 years) and amateur or professional athletes with a training background. To address potential heterogeneity, we conducted pre-planned subgroup analyses based on training status (e.g., trained vs. untrained, athletic vs. recreationally active). Excluded individuals with severe metabolic, cardiovascular, neurological diseases, or other major conditions significantly affecting exercise capacity, inflammation, or oxidative stress levels.

Intervention: Included intervention trials involved oral supplementation with seaweed or microalgae preparations. To ensure conceptual consistency, eligible interventions were operationally defined as whole-biomass preparations or complex algal extracts that retain the natural phytochemical matrix of the algae (including beneficial lipids and secondary carotenoids). These encompassed: (1) Whole algae preparations (e.g., powder, tablets, or capsules of Spirulina or Chlorella); and (2) Algal complex extracts and oleoresins (e.g., brown algal polysaccharides, brown algal polyphenols, and *Haematococcus pluvialis* oleoresin), where the target active compound concentration is typically below 20%. Conversely, we explicitly excluded interventions that represent pure pharmacological entities rather than complex nutritional supplements. Consequently, studies utilizing non-algal sources, chemically synthesized astaxanthin, or single, highly purified molecular isolates (purity > 90%, such as isolated phycocyanin or phycoerythrin) were excluded.

Comparator: Control groups received a placebo or no additional algae-related supplements. The intervention and control groups were required to be comparable regarding exercise tasks and experimental conditions.

Outcomes: Primary outcomes included: (1) Exercise Performance: Maximal oxygen uptake (VO_2_max), time to exhaustion (TTE), WRmax, etc.; (2) Post-exercise Recovery: Muscle damage markers [creatine kinase (CK), lactate dehydrogenase (LDH)], etc.

Secondary outcomes included oxidative stress-related indicators [e.g., malondialdehyde (MDA)].

Study Design: Only human randomized controlled trials (RCTs), including parallel-group and randomized crossover trials, were included. Excluded were animal studies, observational studies (e.g., cohort, case–control), non-randomized trials, and studies using multiple combined interventions where the effect of algae supplementation could not be isolated. The development of these inclusion and exclusion criteria followed the PICOS structure commonly applied in recent systematic reviews on algae-derived nutritional interventions, including the referenced study, to ensure methodological consistency [22].

### 2.4. Data Extraction and Quality Assessment

Two researchers (Y.W., S.L.) independently extracted data using a pre-designed form. For studies that reported change-from-baseline data without the corresponding standard deviation (SD), missing SDs were calculated according to recommended practices in the Cochrane Handbook. When reported, SDs were derived from confidence intervals, *p*-values, or standard errors. For studies where these statistics were also unavailable, the SD of the change was calculated using the formula:

$$SD_{\mathrm{change}}=\sqrt{{\mathrm{SD}}_{\mathrm{baseline}}^{2}+{\mathrm{SD}}_{\mathrm{final}}^{2}-2\cdot r\cdot {\mathrm{SD}}_{\mathrm{baseline}}\cdot {\mathrm{SD}}_{\mathrm{final}}}$$

where SD_baseline_ is the standard deviation at baseline, SD_final_ is the standard deviation at follow-up, and r represents the within-study correlation coefficient between baseline and follow-up measurements. In cases where the correlation coefficient was not reported, r = 0.5 was assumed, reflecting a moderate correlation that is commonly used as a conservative estimate in meta-analyses when within-study correlations are unavailable. This imputation method was applied uniformly to both parallel-group and crossover trials that did not directly report change score SDs or correlations, consistent with the approach used in our previous study [24,25,26]. When feasible, corresponding authors were contacted to obtain missing data prior to imputation.

Risk of bias for each included randomized controlled trial was independently assessed by two reviewers (Y.W., S.L.) using the Cochrane Risk of Bias tool (RoB 2.0) [27]. The following domains were evaluated: (1) bias arising from the randomization process; (2) bias due to deviations from intended interventions; (3) bias due to missing outcome data; (4) bias in measurement of the outcome; and (5) bias in selection of the reported result. Each domain, as well as the overall risk of bias, was judged as “low risk,” “some concerns,” or “high risk” according to the criteria described in the Cochrane Handbook. Discrepancies between reviewers were resolved through discussion or consultation with a third reviewer (C.L.) when necessary. The overall risk-of-bias assessments were considered in the interpretation of pooled results.

### 2.5. Data Synthesis and Statistical Analysis

All outcomes were treated as continuous variables and summarized using standardized mean differences (SMDs) with 95% confidence intervals (CIs). For parallel-group trials, between-group differences in change scores were extracted or calculated. For crossover trials, paired comparisons between intervention and control periods were used.

When crossover studies did not report paired SDs or within-participant correlation coefficients, the SD of the change score was estimated using an assumed correlation coefficient (r = 0.5). Sensitivity analyses were conducted to evaluate the robustness of pooled effect estimates to variations in the assumed correlation coefficient, and the overall conclusions remained unchanged.

Given the anticipated clinical and methodological heterogeneity across studies, all pooled analyses were performed using random-effects models [28]. Heterogeneity was assessed using the *I*^2^ statistic [29]. Statistical significance was set at *p* < 0.05. All analyses were conducted using R 4.5.0 software (R Foundation for Statistical Computing, Vienna, Austria).

### 2.6. Sensitivity Analysis and Publication Bias Assessment

To test the robustness of the pooled results, the following sensitivity analyses were pre-planned and conducted. First, a leave-one-out analysis was performed for primary outcomes. Second, separate subgroup analyses were conducted within feasible ranges based on pre-defined variables (exercise type, algae species, dosage, intervention duration, preparation form, etc.) to explore potential sources of clinical and methodological heterogeneity. We clarify that subgroup analysis and sensitivity analysis are distinct procedures; both were employed as complementary approaches to assess result stability and explore heterogeneity.

When ≥10 studies were included for an outcome, a funnel plot was generated to assess small-study effects and potential publication bias, and Egger’s linear regression test was used to quantitatively test funnel plot asymmetry [30]. In accordance with Cochrane guidance, for outcomes with fewer than 10 studies, we refrained from interpreting funnel plot asymmetry and instead acknowledged the limited power to detect publication bias in these instances.

## 3. Results

### 3.1. Study Selection

**Figure 1:**
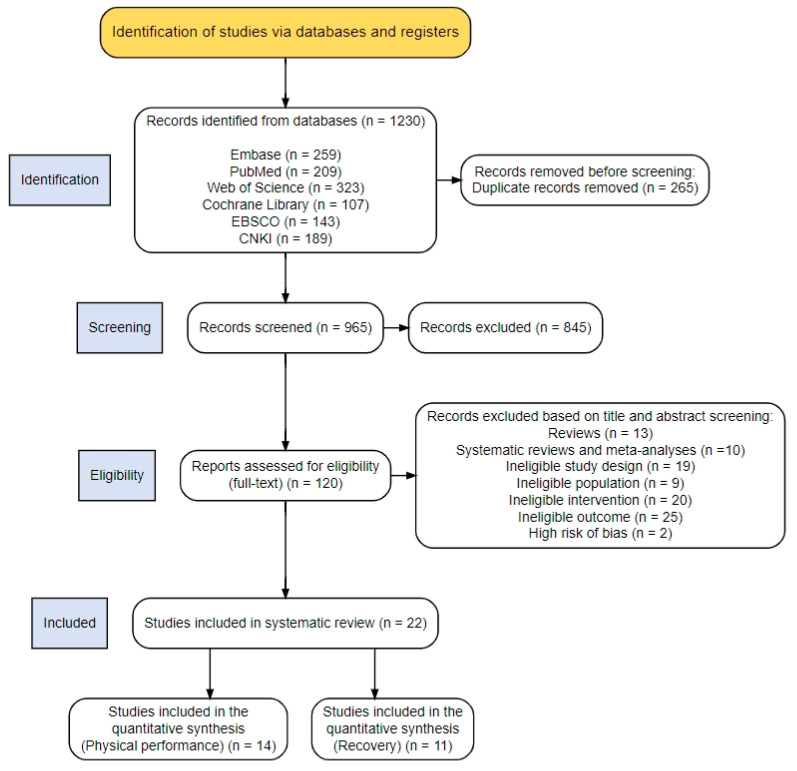
PRISMA flow diagram illustrating the identification, screening, eligibility assessment, and inclusion of studies in this systematic review and meta-analysis.

The initial database search identified 1230 records, including 259 from Embase, 209 from PubMed, 323 from Web of Science, 107 from Cochrane Library, 143 from EBSCO, and 189 from CNKI. After removal of 265 duplicates, 965 records remained for title and abstract screening. Of these, 845 were excluded due to irrelevance to the predefined population, intervention, or outcomes criteria. A total of 120 full-text articles were assessed for eligibility. Following full-text review, 22 RCTs met the inclusion criteria and were included in the qualitative synthesis. Among these, 14 studies contributed data to the meta-analysis of performance outcomes, and 11 contributed data to recovery-related outcomes. The study selection process is illustrated in Figure 1.

### 3.2. Characteristics of Included Studies

All 22 included studies were RCTs. Participants were predominantly healthy adults or physically active individuals, including university students, recreationally trained individuals, and a smaller proportion of competitive athletes. Most studies were single-center studies with relatively small sample sizes. The interventions comprised whole-algae supplements (e.g., *Spirulina*, *Chlorella*), brown algae extracts, and algal -derived astaxanthin. Supplements were primarily administered in capsule, tablets, or powdered beverage form. Intervention durations ranged from acute single-dose protocols to chronic supplementation lasting several weeks. Control groups typically received placebo preparations or isoenergetic non-algal comparators.

Exercise protocols included continuous aerobic exercise, high-intensity interval training (HIIT), and resistance training. Reported outcomes encompassed measures of exercise performance (e.g., VO_2_max, time to exhaustion, WRmax, and time-trial performance), biomarkers of muscle damage and recovery (e.g., CK, LDH, DOMS, etc.), and indices of oxidative stress (e.g., MDA, thiobarbituric acid reactive substances (TBARS), advanced oxidation protein products (AOPP), sulfhydryl (SH) groups, and various antioxidant enzyme activities). Detailed characteristics of the included studies are summarized in Table 1.

### 3.3. Risk of Bias Assessment

**Figure 2:**
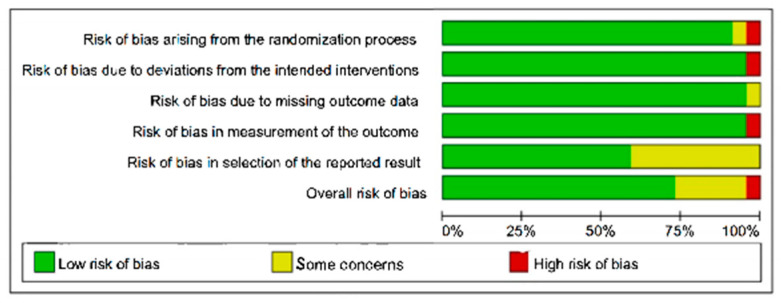
Risk of bias summary for all included randomized controlled trials based on the RoB 2 tool. The figure presents the proportion of studies classified as low risk (green), some concerns (yellow), or high risk (red) across the five RoB 2.0 domains: randomization process, deviations from intended interventions, missing outcome data, measurement of outcomes, and selection of the reported results. Overall, most studies demonstrated low risk of bias across major domains, with some concerns primarily related to selective reporting and outcome measurement.

Risk of bias was evaluated using the Cochrane Risk of Bias 2 (RoB 2) tool across five domains for all 22 included trials (Figure 2). Overall, most studies were judged to have a low risk of bias in the domains of missing outcome data and selection of reported results.

However, several studies presented limitations related to the randomization process, allocation concealment, blinding of participants and personnel, and outcome measurement. These methodological shortcomings resulted in ratings of “some concerns” in multiple domains for a considerable proportion of trials. A small number of earlier studies did not report sample size calculations or prospective trial registration, increasing the potential risk of reporting bias. Overall, the methodological quality of the included trials was considered acceptable. Nevertheless, these limitations should be taken into account when interpreting the magnitude and robustness of the pooled effect estimates.

### 3.4. Overall Effects on Primary Outcomes

**Figure 3:**
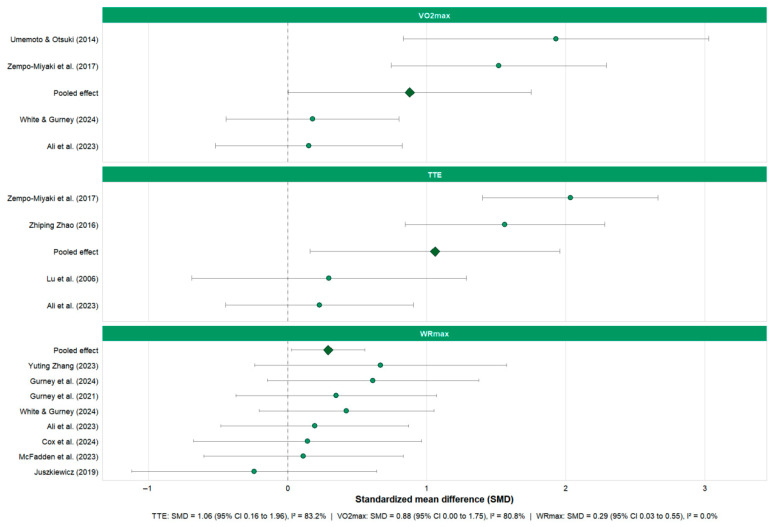
Forest plots of the effects of seaweed and microalgae supplementation on aerobic performance outcomes [32,35,36,39,44,45,46,47,51,52,53,54]. Forest plots present pooled standardized mean differences (SMD) and 95% confidence intervals (CIs) for three key performance indicators: maximal oxygen uptake (VO_2_max), time to exhaustion (TTE), and maximal workload/peak power output (WRmax). Algae supplementation was associated with moderate improvements in VO_2_max (pooled SMD = 0.88, 95% CI: 0.00–1.75; *I*^2^ = 80.8%) and TTE (pooled SMD = 1.06, 95% CI: 0.16–1.96; *I*^2^ = 83.2%). A smaller but statistically significant effect was observed for WRmax (pooled SMD = 0.29, 95% CI: 0.03–0.55; *I*^2^ = 0%), and diamonds indicate pooled effects derived from random-effects models, and circles denote individual study estimates.

**Figure 4:**
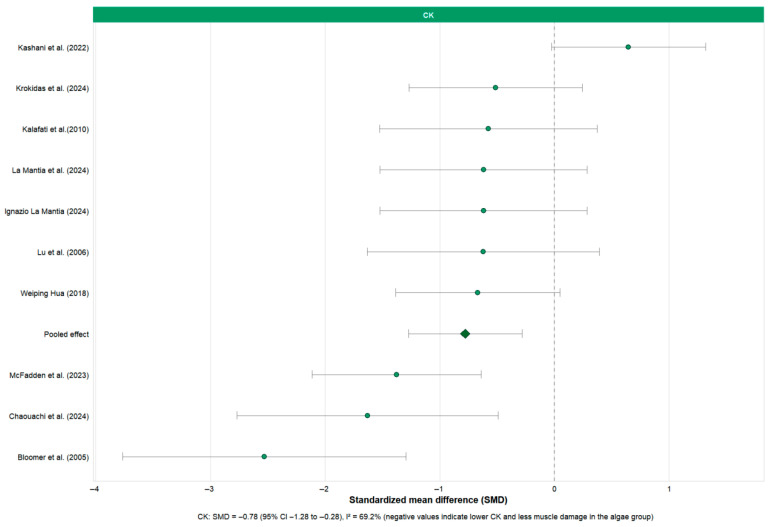
Forest plot of the effects of algae supplementation on creatine kinase (CK) [31,32,33,41,42,45,48,49,50]. The forest plot displays pooled SMD with 95% CIs for post-exercise CK concentrations. The overall pooled estimate (random-effects model) demonstrates a significant reduction in CK levels following algae supplementation compared with control (SMD = −0.78, 95% CI: −1.28 to −0.28, *I*^2^ = 69.2%), suggesting a potential attenuation of exercise-induced muscle damage.

**Figure 5:**
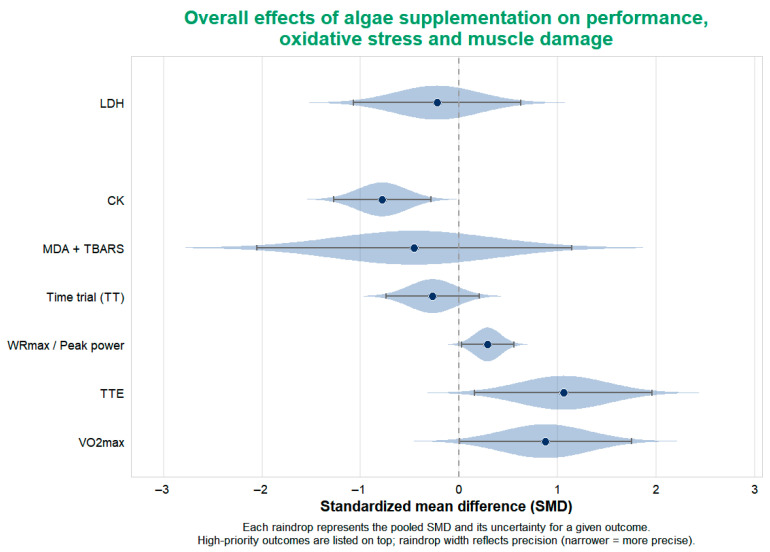
Rainforest plot of pooled effects of algae supplementation across performance and recovery outcomes. The Rainforest plot illustrates the distribution of pooled SMDs across performance, oxidative stress, and muscle damage markers. Raindrop width reflects study precision, with narrower distributions indicate greater precision. Outcomes are displayed according to predefined priority. Positive SMD values represent improvements in performance-related outcomes, whereas negative SMD values indicate reductions in muscle damage or oxidative stress markers.

Pooled meta-analyses were first conducted for physical performance outcomes, including VO_2_max, TTE, WRmax, TT (Figure 3). Subsequently, pooled analyses were performed for post-exercise muscle damage and recovery indicators, including CK and LDH (Figure 4). Forest plots were generated to illustrate the pooled effect sizes and corresponding confidence intervals for each primary outcome. Additionally, a rainforest plot was constructed to visualize the overall distribution and dispersion of effect sizes across performance and recovery endpoints (Figure 5).

#### 3.4.1. Aerobic Performance Indicators (VO_2_max, TTE, WRmax, TT)

As shown in Figure 3, the overall effect direction of algae supplementation on aerobic-related indicators was generally favorable, but the strength, consistency, and practical meaning of effects varied considerably across outcomes.

For VO_2_max, the random-effects model yielded a moderate pooled effect estimate; however, the lower bound of the 95% confidence interval approached the null value (SMD = 0.88, 95% CI: 0.00–1.75), and between-study heterogeneity was substantial (*I*^2^ = 80.8%). These findings indicate that, although an overall tendency toward improved aerobic capacity was observed, the precision of the estimate was limited and the magnitude of benefit varied considerably across studies. Therefore, the pooled result should be interpreted as suggestive rather than definitive evidence for a VO_2_max-enhancing effect of algae supplementation.

For time to exhaustion (TTE), algae supplementation was associated with a statistically significant pooled effect (SMD = 1.06, 95% CI: 0.16–1.96). Nevertheless, heterogeneity was extremely high (*I*^2^ = 83.2%), indicating marked variability in effect magnitude across studies. As such, while the direction of effect consistently favored supplementation, the size of the benefit appears highly context-dependent and should not be assumed to be generalizable across different populations, exercise protocols, or algae preparations.

For WRmax, a small but statistically significant pooled effect was observed (SMD = 0.29, 95% CI: 0.03–0.55), with no detectable heterogeneity (*I*^2^ = 0%). Although this suggests a consistent direction of effect across studies, the magnitude of improvement was modest. Therefore, the practical relevance of this finding, particularly in competitive performance settings, remains uncertain.

To reflect real competition scenarios, time-trial (TT) performance was analyzed separately (Section S2 Figure S1). Based on three trials in the random-effects model, the overall effect of algae supplementation on TT performance was close to zero (SMD = −0.27, 95% CI: −0.74–0.21, *I*^2^ = 0.0%). Current evidence is insufficient to support a consistent improvement in time-trial performance with algae supplementation.

#### 3.4.2. Post-Exercise Recovery and Muscle Damage Indicators (CK, LDH)

To assess the effect of algae supplementation on exercise-induced muscle damage, meta-analyses were conducted for CK and LDH (Figure 4 and Section S3 Figure S2).

For creatine kinase (CK), the pooled analysis showed a moderate reduction following algae supplementation (SMD = −0.78, 95% CI: −1.28 to −0.28), accompanied by substantial heterogeneity (*I*^2^ = 69.2%). Importantly, this estimate was influenced by a limited number of studies reporting relatively large effects. Although sensitivity analyses did not materially alter the direction of the pooled result, the presence of between-study variability and the small number of contributing trials suggest that the magnitude of CK reduction should be interpreted cautiously.

For LDH (Section S4 Figure S3), the random-effects model based on four trials showed a small, non-significant effect (SMD = −0.22, 95% CI: −1.07–0.62, *I*^2^ = 74.8%). Current evidence is insufficient to conclude that algae supplementation consistently reduces LDH levels post-exercise.

Overall, the CK results provide relatively consistent quantitative support for “algae supplementation helping to reduce exercise-induced muscle damage,” while evidence for LDH is more scattered and less conclusive. Both are affected by significant heterogeneity and should be interpreted in the context of specific sports and supplementation strategies.

#### 3.4.3. Oxidative Stress Indicators

For the lipid peroxidation marker MDA, the initial random-effects model showed that astaxanthin supplementation did not have a significant overall effect on exercise-induced MDA levels (SMD = −0.17, 95% CI: −1.34–1.00, *p* = 0.78), with extremely high between-study heterogeneity (*I*^2^ = 89%) (forest plot in Section S5 Figure S4). Leave-one-out sensitivity analysis revealed that the study by Tsao et al. had a substantial influence on the pooled effect and heterogeneity. After excluding this study, the pooled effect shifted to a moderate-to-large positive direction (SMD = 0.53, 95% CI: −0.03–1.09, *p* = 0.07), and heterogeneity decreased to moderate (*I*^2^ = 50%), but it still did not reach statistical significance. In summary, the limited number of astaxanthin trials yields inconsistent results, and it is currently difficult to conclude that it stably reduces exercise-related MDA levels.

#### 3.4.4. Overall Distribution in Rainforest Plot (Figure 5)

To compare the effect sizes of primary outcomes in a single view, a rainforest plot was created (Figure 5), displaying the pooled effects for VO_2_max, TTE, WRmax, and CK. The plot shows:

(1) The summary effect points for VO_2_max and TTE are positioned to the right of the zero line, with a more pronounced shift for TTE.
(2) WRmax also leans to the right, but with a smaller effect value.
(3) The effect point for CK is on the left side of the zero line, indicating an overall decreasing trend for this muscle damage marker.

This distribution aligns with the individual analyses: algae supplementation shows the most prominent advantage in improving endurance-type performance (especially TTE); the magnitude of improvement in peak power is smaller; and there is a relatively consistent protective effect on CK regarding muscle damage.

### 3.5. Subgroup Analyses

#### 3.5.1. Subgroup Analysis for Performance Outcomes

**Figure 6:**
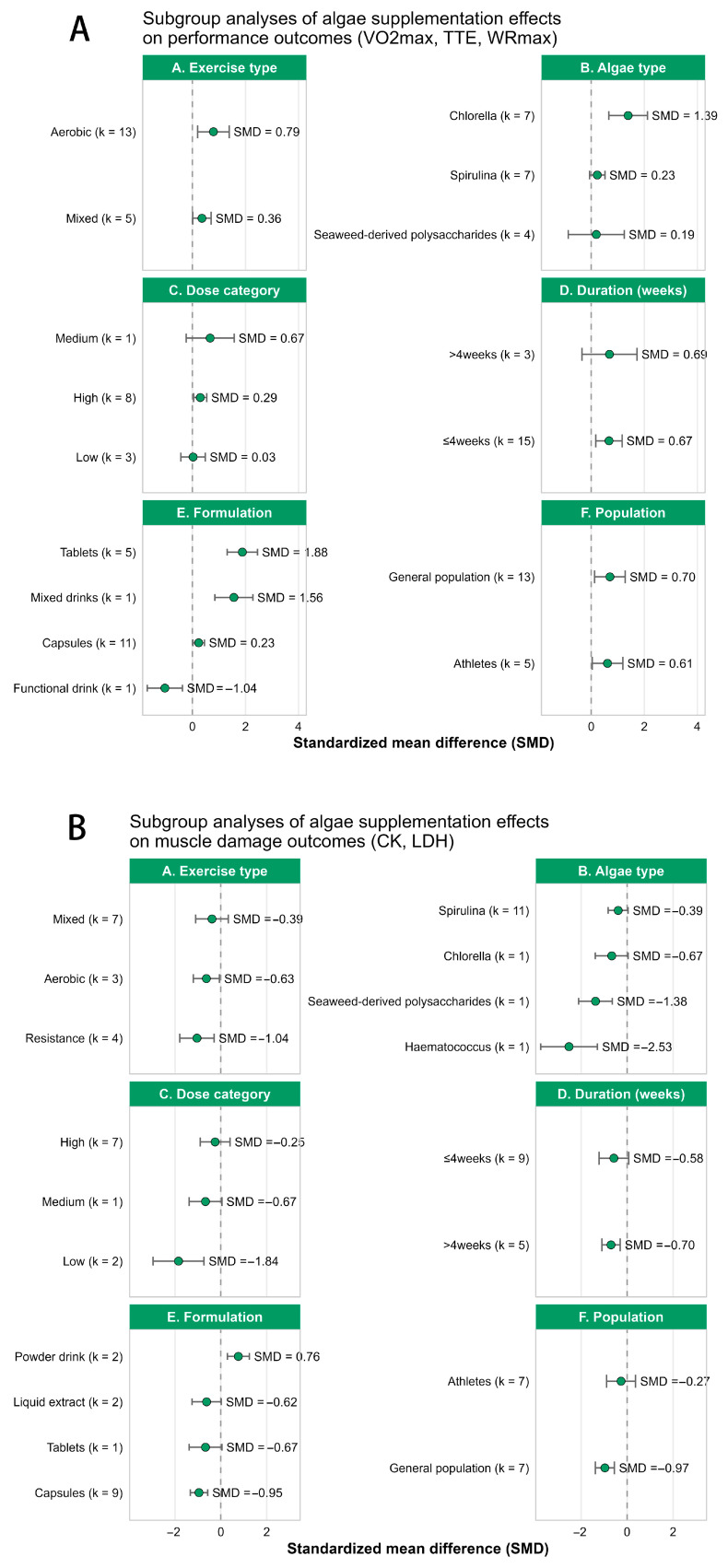
Subgroup analyses of the effects of algae supplementation on performance and recovery outcomes. (**A**) Subgroup analysis of performance outcomes (VO_2_max, TTE, WRmax) based on exercise type, algae type, dose category, intervention duration, formulation and population. (**B**) Subgroup analysis of muscle damage and recovery outcomes (CK, LDH) based on exercise type, algae type, dose category, intervention duration, and formulation.

Subgroup results for performance outcomes (VO_2_max, TTE, WRmax) are shown in Figure 6A. Overall, most subgroup-specific effect sizes favored algae supplementation, although the magnitude varied across categories.

A clearer pattern emerged for exercise type and algae type. The aerobic subgroup showed a larger effect than the mixed subgroup (SMD = 0.79 vs. 0.36), suggesting that algae supplementation may be more beneficial in endurance-oriented settings. Among algae types, Chlorella showed the largest effect estimate (SMD = 1.39), whereas Spirulina and seaweed-derived polysaccharides showed much smaller effects (SMD = 0.23 and 0.19, respectively).

For dose and duration, the pattern was less distinct. Medium- and high-dose interventions were associated with favorable effects, whereas the low-dose subgroup showed little benefit (SMD = 0.03). Likewise, the pooled effects were similar between interventions lasting more than 4 weeks and those lasting 4 weeks or less (SMD = 0.69 vs. 0.67), suggesting no obvious duration-related gradient in the current evidence.

Some variation was also evident by formulation and population. Tablets showed a relatively large effect (SMD = 1.88), while capsules showed a smaller effect (SMD = 0.23); notably, the functional drink subgroup showed an opposite direction (SMD = −1.04). Beneficial effects were observed in both the general population and athletes, with a slightly larger estimate in the general population (SMD = 0.70 vs. 0.61). Overall, these findings suggest that performance-related benefits may be more evident in aerobic settings and in studies using Chlorella or tablet-based preparations, although several subgroup estimates were based on few studies and should be interpreted cautiously.

#### 3.5.2. Subgroup Analysis for Muscle Damage and Recovery Outcomes

Subgroup results for muscle damage and recovery outcomes are presented in Figure 6B. Overall, most subgroup-specific effect sizes favored algae supplementation, although the magnitude varied across categories.

The most notable pattern was seen across exercise types. The largest reduction was observed in resistance exercise (SMD = −1.04), followed by aerobic exercise (SMD = −0.63) and mixed exercise (SMD = −0.39), suggesting that the recovery-related benefits of algae supplementation may be more evident under conditions involving greater mechanical load and muscle damage.

Differences were also observed across algae types. Spirulina showed a modest beneficial effect (SMD = −0.39), whereas Chlorella showed a somewhat larger estimate (SMD = −0.67). Even larger effects were observed for seaweed-derived polysaccharides (SMD = −1.38) and *Haematococcus pluvialis*-derived astaxanthin (SMD = −2.53), although these estimates were each derived from a single study and therefore should be interpreted with caution.

No clear dose–response pattern was evident. The low-dose subgroup showed the largest point estimate (SMD = −1.84), whereas the high-dose subgroup showed a smaller effect (SMD = −0.25). Similarly, the pooled effects were broadly comparable between interventions lasting 4 weeks or less and those lasting more than 4 weeks (SMD = −0.58 vs. −0.70).

By formulation, capsules, tablets, and liquid extract all showed beneficial effects, whereas powder drink showed an opposite direction (SMD = 0.76). The effect was also more favorable in the general population than in athletes (SMD = −0.97 vs. −0.27). Overall, these findings suggest that the recovery-related benefits of algae supplementation may be more evident in resistance-based exercise and in the general population, although several subgroup estimates were derived from very few studies and should be interpreted cautiously.

#### 3.5.3. Meta-Regression and Dose–Duration Effects

**Figure 7:**
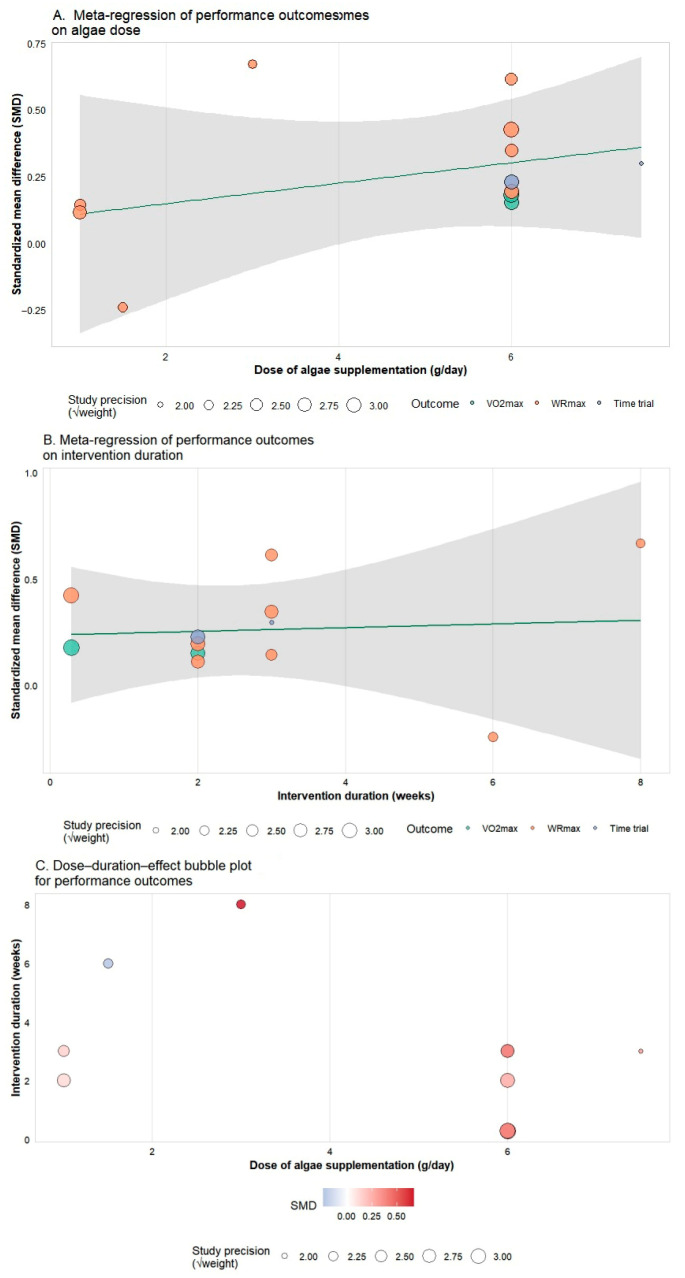
Meta-regression analyses of algae supplementation dose and intervention duration on performance outcomes. (**A**) Meta-regression of standardized mean difference (SMD) for performance outcomes on algae dose (g/day). (**B**) Meta-regression of SMD for performance outcomes on intervention duration (weeks). In panels A and B, the solid lines represent the fitted meta-regression models, the shaded areas indicate the 95% confidence intervals, and the bubble colors denote specific outcome types (e.g., VO_2_max, WRmax, Time trial). (**C**) Bubble plot illustrating the combined effect of dose and duration on performance outcomes, where the color gradient represents the specific SMD value. For all panels, the size of the bubble is proportional to the study weight (precision).

Meta-regression analyses were performed to explore whether algae dose or intervention duration contributed to the variability in performance outcomes (Figure 7A–C). A slight positive trend was observed between supplementation dose and effect size, with higher doses—particularly around 6 g/day—showing somewhat larger improvements in VO_2_max, TTE, and WRmax. Similarly, intervention duration displayed a mild upward trend, suggesting that longer supplementation periods may be associated with greater benefits, although neither relationship reached statistical significance. When dose and duration were examined jointly in a combined bubble plot, studies using moderate-to-high doses over multi-week periods tended to show more favorable performance responses, whereas lower doses generally produced smaller effects. Overall, these findings indicate no clear linear dose–response or duration–response pattern, but they do suggest that adequate dosing and sustained supplementation may be important for achieving meaningful ergogenic effects.

### 3.6. Sensitivity Analysis and Publication Bias

**Figure 8:**
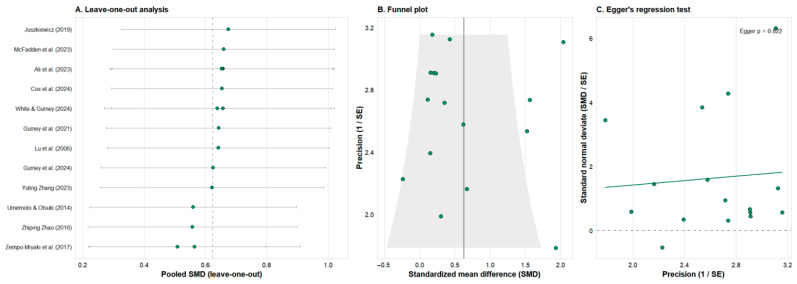
Sensitivity analysis and publication bias assessment for performance outcomes (VO_2_max, TTE, WRmax). (**A**) Leave-one-out sensitivity analysis showing the influence of individual studies on the pooled effect size (SMD) and heterogeneity (*I*^2^) [32,35,36,39,44,45,46,47,51,52,53,54]. Each line represents the result of removing one study, confirming the robustness of the meta-analysis. (**B**) Funnel plot for assessing small-study effects and potential publication bias. Asymmetry may indicate publication bias. (**C**) Egger’s regression test for funnel plot asymmetry. A significant deviation from zero (*p* < 0.10) suggests the presence of publication bias.

Leave-one-out sensitivity analysis for primary outcomes (Figure 8A and Section S6 Figure S5) showed that the direction and magnitude of effect sizes for most outcomes did not change substantively, indicating overall result robustness. Heterogeneity for certain indicators (e.g., MDA, comprehensive antioxidant enzyme activity) was mainly driven by a few studies inconsistent with the overall trend; excluding these studies improved the pooled effect size and I^2^ to some extent, but statistical significance remained limited.

For outcomes with ≥10 included studies, funnel plots (Figure 8B and Section S7 Figure S6) did not show significant asymmetry, and Egger’s regression tests (Figure 8C and Section S8 Figure S7) did not indicate clear small-study effects or publication bias. It should be noted that for some outcomes (e.g., LDH), the limited number of studies reduces the statistical power of bias tests, and the possibility of “positive results being more likely to be published” cannot be entirely ruled out; conclusions should be interpreted with caution.

### 3.7. Multi-Indicator Integrated Visualization Analysis

**Figure 9:**
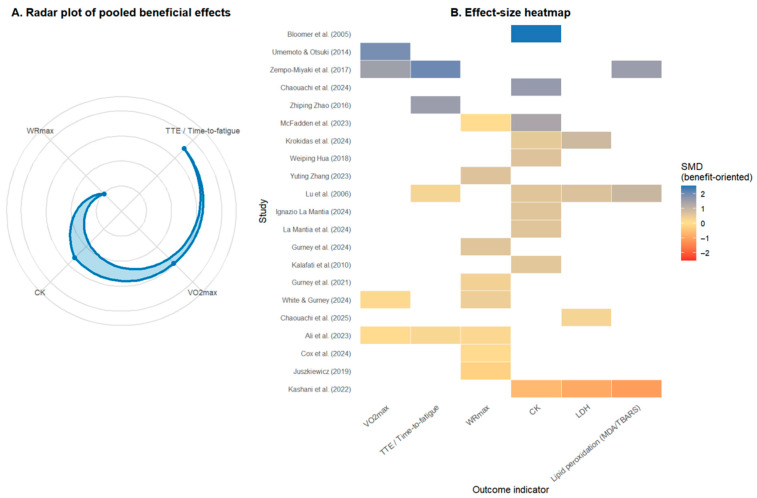
Multidimensional visualization of algae supplementation effects [31,32,33,35,36,39,41,42,43,44,45,46,47,48,49,50,51,52,53,54]. (**A**) Radar plot showing pooled beneficial effects across key performance and recovery outcomes. Each axis represents a different outcome, and the plotted area indicates the overall effect magnitude across studies. (**B**) Effect-size heatmap of standardized mean differences (SMD) across studies and outcomes. Colors represent effect direction and magnitude (blue = beneficial, red = harmful), illustrating the consistency and variability of algae effects on different performance and recovery indicators.

To visually present the comprehensive impact of algae supplementation across multiple outcomes and the variability between studies, radar plots and effect size heatmaps were constructed (Figure 9).

The radar plot, using benefit-oriented SMD, shows TTE and VO_2_max positioned towards the outer edges of the polygon, with overall effect sizes in the small-to-moderate range, suggesting that algae supplementation’s enhancing effect on endurance-related performance is relatively more prominent. In contrast, the radii corresponding to WRmax and CK are shorter, reflecting only small beneficial effects, indicating that while improvement trends exist for peak power and muscle damage mitigation, the magnitudes are weaker than for endurance indicators.

The effect size heatmap reveals inconsistencies across different trials from a two-dimensional “study × indicator” perspective. Overall, most studies showed light to moderate blue shading for VO_2_max, TTE, or WRmax, corresponding to small-to-moderate beneficial effects. Some studies showed near-neutral shading, indicating not all trials observed significant performance improvements. For recovery-related indicators, CK and LDH mostly showed light yellow or light blue shading, indicating an overall trend of slight reduction in muscle enzymes. At the same time, individual studies showed warm-colored shading for CK, LDH, or MDA/TBARS, suggesting that algae supplementation’s improvement of muscle damage or lipid peroxidation may be unstable or even slightly reversed in certain contexts.

In summary, the results from the radar plot and heatmap corroborate the meta-analyses and subgroup analyses: existing evidence relatively consistently supports the advantage of algae supplementation in aerobic endurance performance, while the benefits for muscle damage and oxidative stress recovery are generally moderate and dependent on specific populations and intervention protocols.

## 4. Discussion

### 4.1. Main Findings

This systematic review and meta-analysis suggests that seaweed and microalgae supplementation may exert conditional and outcome-specific effects on exercise performance and post-exercise recovery, rather than uniform benefits across all endpoints.

With respect to aerobic exercise capacity, pooled analyses indicated moderate average effects on VO_2_max and time to exhaustion (TTE); however, these findings were accompanied by substantial uncertainty and considerable between-study heterogeneity. In particular, the confidence interval for VO_2_max approached the null value, and heterogeneity for TTE was high, indicating that the magnitude and reliability of these effects vary markedly across studies. Individual trials have reported prolonged TTE following algae supplementation, such as a study in which four weeks of Spirulina intake was associated with extended high-intensity running performance alongside altered substrate utilization patterns [33]. While such findings suggest a potential link between algae supplementation and improved metabolic efficiency during endurance exercise, they should be interpreted as context-dependent observations rather than definitive evidence of a consistent ergogenic effect. Similarly, improvements in submaximal physiological responses (e.g., heart rate, blood lactate, or recovery kinetics) have been reported in some studies [55], but these responses were not uniformly observed across the literature.

Regarding post-exercise recovery, pooled results suggested a moderate reduction in creatine kinase (CK) following algae supplementation; however, this effect was characterized by substantial heterogeneity and appeared to be influenced by a subset of studies reporting relatively large effects. Experimental trials have shown attenuated increases in CK and inflammatory markers such as C-reactive protein after high-intensity exercise in Spirulina-supplemented participants [43], indicating a possible protective role against exercise-induced muscle damage and inflammation. Nevertheless, the variability in study designs, exercise protocols, and supplementation strategies limits the generalizability of these findings, and the magnitude of recovery-related benefits remains uncertain.

Taken together, the present evidence indicates that the effects of algae supplementation are not universal and are likely moderated by factors such as algae species, dosage, intervention duration, participant training status, baseline nutritional status, and exercise modality. Although pooled estimates point toward potential benefits for selected performance and recovery outcomes, particularly TTE and CK, the practical significance and robustness of these effects—especially for VO_2_max—remain unclear. Overall, algae supplementation may represent a potentially supportive sports nutrition strategy under specific conditions, but confirmation of its efficacy will require larger, well-controlled trials with standardized outcome measures.

Moreover, it is essential to bridge the gap between statistical significance and practical relevance. While our pooled analysis revealed a substantial positive shift in TTE (SMD = 1.06), the results for maximal workload (WRmax, SMD = 0.29) were modest, and time-trial (TT) performance—the metric most reflective of real-world competitive success—showed no significant overall benefit (SMD = −0.27). This discrepancy implies that while algae supplementation efficiently enhances submaximal metabolic economy and prolongs the capacity to sustain effort in open-loop tasks (TTE), it may lack the rapid ergogenic punch required to meaningfully alter high-intensity, closed-loop competitive performance (TT or 1-RM strength). Therefore, coaches and athletes should view algae primarily as a long-term foundational supplement to tolerate higher training volumes and expedite recovery, rather than an acute performance-enhancing aid for race day.

### 4.2. Mechanisms and Application of Aerobic Performance Improvement

#### 4.2.1. Antioxidant Effects Delaying Fatigue

As quantitatively evidenced by our pooled analysis (Figure 3), algae supplementation significantly prolonged TTE (SMD = 1.06) and provided a suggestive benefit for VO_2_max (SMD = 0.88). A primary mechanism underlying this substantial endurance enhancement may be attributed to the potent antioxidant properties of algae. Strenuous exercise generates excess reactive oxygen species (ROS), leading to muscle fatigue and cellular damage. Antioxidant components abundant in seaweed and microalgae can enhance the body’s antioxidant capacity, help clear excessive ROS, and maintain intracellular redox balance [56,57]. This can delay the onset of fatigue caused by oxidative stress to some extent, improving tolerance to high-intensity exercise. For instance, one study reported that Spirulina supplementation significantly increased resting and post-exercise glutathione (GSH) levels while suppressing exercise-induced increases in lipid peroxidation markers like malondialdehyde and thiobarbituric acid reactive substances [58]. As these oxidative damage markers did not rise significantly, subjects exhibited better endurance persistence. Thus, algae may reduce cumulative oxidative damage during exercise through potent antioxidant effects, thereby delaying fatigue and enhancing aerobic performance.

#### 4.2.2. Promotion of Nitric Oxide Release and Blood Flow/Oxygen Supply

Active components in algae may also improve aerobic capacity through vasodilation mechanisms. Phycocyanin in Spirulina has been shown in animal studies to increase nitric oxide (NO) levels and enhance endothelial nitric oxide synthase activity, promoting vasodilation [59]. In humans, Chlorella supplementation also shows indications of enhancing the NO pathway: healthy men consuming 6 g of Chlorella daily for 4 weeks showed significantly reduced arterial stiffness, suggesting improved peripheral vascular compliance. More importantly, a significant increase in peak oxygen uptake was observed under the same protocol, with the improvement in VO_2_max partially attributed to NO-mediated blood flow improvement. Although direct human studies measuring algae-induced increases in NO bioavailability are currently limited, these results support the potential mechanism of algae improving muscle blood and oxygen supply to enhance aerobic endurance. Enhanced blood perfusion means working muscles receive more adequate oxygen and nutrients, enabling maintenance of higher power output during prolonged exercise.

#### 4.2.3. Increasing Hemoglobin Levels and Oxygen-Carrying Capacity

Spirulina is rich in highly bioavailable iron and protein, making it an excellent source of hematopoietic raw materials [5,60]. Unlike many plant foods, Spirulina lacks phytates and oxalates that interfere with iron absorption, and its iron is easily absorbed. Studies have found that even in non-anemic healthy athletic populations, Spirulina supplementation can induce an upward trend in hemoglobin (Hb) concentration. Since hemoglobin is the key carrier for transporting oxygen from lungs to muscles, theoretically, increased Hb could improve oxygen delivery efficiency and enhance aerobic metabolic capacity during exercise. Some researchers have reported improvements in exercise endurance indicators alongside increased Hb with Spirulina supplementation, suggesting a possible association. Therefore, for athletes with relatively low baseline iron stores, the abundant iron and vitamins in Spirulina may be particularly beneficial for correcting subclinical anemia, thereby improving aerobic performance. However, this mechanism requires further direct evidence, and future research should focus on different genders and iron status populations.

#### 4.2.4. Improving Substrate Metabolism and Energy Utilization

Algae supplementation can also enhance aerobic endurance by modulating substrate utilization patterns during exercise. In endurance sports, delaying glycogen depletion is crucial for sustained performance. Microalgae like Spirulina can promote fat oxidation and reduce reliance on glycolysis to some extent, achieving a “glycogen-sparing effect.” The classic study by Kalafati et al. [33] showed that the Spirulina group had a 10.3% lower carbohydrate oxidation rate and a 10.9% higher fat oxidation rate during 2 h of moderate-intensity running compared to the placebo group, ultimately extending time to exhaustion in a subsequent sprint phase. Due to improved fat oxidation efficiency, subjects experienced less lactate accumulation in the later stages of exercise, delaying fatigue onset. This optimized substrate metabolic state benefits performance in prolonged aerobic tasks. Furthermore, some studies observed that Spirulina supplementation reduced post-exercise peak blood lactate levels and promoted lactate clearance after high-intensity exercise, suggesting improved metabolic homeostasis, allowing muscles to endure less metabolic stress under the same workload. In summary, by promoting fat oxidation and reducing accumulation of anaerobic byproducts, algae enhance metabolic economy, offering practical value for endurance events like marathons and long-distance cycling. Coaches and athletes may consider incorporating algae supplements during training and competition periods to optimize energy utilization and extend the duration of high-level output.

**Figure 10:**
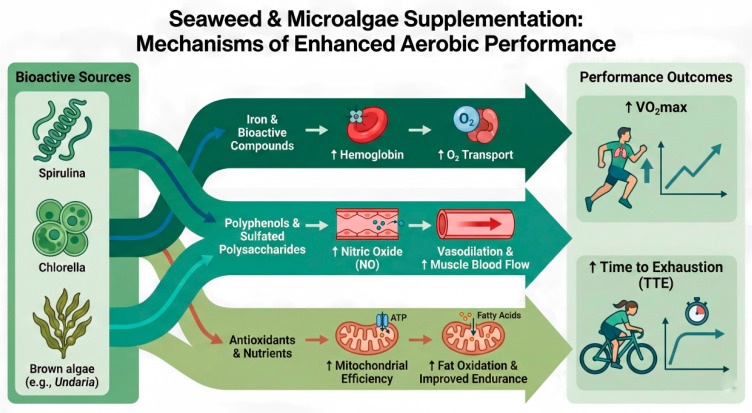
Mechanisms through which Spirulina, Chlorella, and brown algae-derived compounds may enhance aerobic exercise capacity. Key pathways include: (1) increased hemoglobin and oxygen transport; (2) polyphenol- and polysaccharide-induced nitric oxide (NO) production and improved muscle blood flow; and (3) mitochondrial support and enhanced fat oxidation. These integrated actions contribute to observed improvements in VO_2_max and time to exhaustion.

Taken together, these interrelated mechanisms indicate that algae-derived bioactive compounds enhance oxygen transport, vascular function, mitochondrial efficiency, and substrate utilization, thereby improving VO_2_max and prolonging time to exhaustion, as summarized in Figure 10.

### 4.3. Significance and Mechanisms of Muscle Damage and Recovery

#### 4.3.1. Reducing Oxidative and Inflammatory Damage

Our meta-analysis demonstrated a moderate but clinically relevant reduction in post-exercise CK concentrations (SMD = −0.78) following algae supplementation. This attenuation of muscle damage markers is fundamentally linked to the capacity of algae-derived bioactive compounds to blunt oxidative and inflammatory damage at the cellular level. High-intensity or prolonged exercise causes microscopic damage to skeletal muscle, triggering inflammatory responses and oxidative stress, which are primary causes of delayed-onset muscle soreness and functional decline [61]. Antioxidants and anti-inflammatory nutrients abundant in algae can mitigate these harmful processes at their source [62]. Spirulina supplementation can reduce post-exercise ROS attack on muscle cell membranes, preventing excessive generation of lipid peroxides, while also modulating immune responses and reducing the release of pro-inflammatory mediators [63]. In an RCT involving rugby players, approximately 5.7 g of Spirulina daily for 7 weeks significantly suppressed increases in various damage markers after exhaustive exercise: the placebo group showed significant spikes immediately post-exercise in F2-isoprostanes (a lipid peroxidation marker), CRP, and CK, while these markers remained largely stable in the Spirulina group. This indicates Spirulina effectively prevented cell membrane lipid oxidation and muscle tissue micro-damage induced by intense contact sport. Importantly, inflammation levels were also lower in the Spirulina group, suggesting synergistic antioxidant and anti-inflammatory effects, reducing the total damage requiring repair in muscle tissue post-exercise. By decreasing the initial degree of damage, algae create a better internal environment for subsequent muscle fiber regeneration and functional recovery, which is significant for athletes requiring frequent high-intensity training—less cumulative damage means lower risks of overtraining and injury.

#### 4.3.2. Promoting Recovery and Maintaining Exercise Capacity

The role of algae in shortening post-exercise recovery time and maintaining subsequent performance holds significant practical value. If muscle damage and inflammation subside more quickly, athletes can return to a state ready for training and competition sooner. Algae supplements like Spirulina accelerate the process of restoring the body to balance from a stressed state through the mechanisms described above. For example, the study by Chaouachi et al. showed that 24 h after high-intensity interval running, athletes in the Spirulina group not only did not maintain high levels of CK and CRP like the placebo group but showed significant decreases below baseline levels. In contrast, these markers remained significantly higher than resting levels in the placebo group at 24 h, indicating incomplete muscle repair. The effect of Spirulina accelerating the decline of damage markers implies faster muscle tissue repair and functional restoration. Faster physiological recovery reduces the time needed between training sessions, allowing athletes to perform high-quality training more frequently without being forced to reduce intensity due to unresolved fatigue and micro-damage. Therefore, some researchers suggest that for athletic populations with heavy training loads and insufficient daily dietary antioxidant intake, Spirulina could be considered a functional supplement to reduce cumulative oxidative and inflammatory stress from repeated training and competition, thereby minimizing performance decline and accelerating post-event recovery.

It is important to note that not all studies report clear effects of algae on promoting recovery. For instance, one trial using upper limb eccentric exercise to induce damage found that 15 days of Spirulina supplementation did not significantly improve muscle strength recovery or subjective muscle soreness compared to placebo. This suggests that the benefits of algae for recovery may vary depending on the type of damage, assessment indicators, and individual differences. Overall, most evidence supports that algae promote recovery by reducing exercise-induced muscle damage, but their long-term effects on functional recovery and performance require further validation.

### 4.4. Differences Among Algae Preparations and Intervention Protocols

#### 4.4.1. Conceptual Heterogeneity of Algae Supplementation and Its Implications for Interpretation

It is essential to acknowledge that the term “algae supplementation” encompasses a highly heterogeneous set of interventions, including whole-organism preparations, complex algal extracts (e.g., brown-algal polysaccharides, polyphenols), and specific algal-derived compounds (e.g., astaxanthin from *Haematococcus pluvialis*). These substances differ substantially in their bioactive profiles, proposed mechanisms of action, dosages, and target populations. For instance, Spirulina is rich in phycocyanin, iron, and γ-linolenic acid, potentially supporting erythropoiesis and antioxidant defense; Chlorella provides chlorophyll, lutein, and immune-modulating polysaccharides; brown-algal extracts often deliver sulfated polysaccharides with anti-inflammatory properties; and astaxanthin is a potent carotenoid antioxidant. Such compositional and mechanistic diversity implies that the pooled effects presented in this meta-analysis should be interpreted as an aggregate signal of a heterogeneous class of bioactive supplements, rather than a uniform effect of a single compound. While subgroup analyses by algae type provided some differentiation, the limited number of studies within each category precludes definitive conclusions about superiority or specificity. This conceptual heterogeneity is a key limitation of the current evidence base and suggests that future research should adopt more standardized, head-to-head comparisons of different algae types within the same trial to clarify their distinct roles in sports nutrition.

#### 4.4.2. Biological Profiles and Variations in Supplementation Strategies

Spirulina and Chlorella are currently the most extensively researched algae supplements in sports nutrition. They differ in biological characteristics and nutritional composition, which is reflected in their intervention effects. Spirulina is a filamentous cyanobacterium without a rigid cellulose wall, allowing efficient human digestion after harvesting and drying. Chlorella is a green alga with a thick cell wall that typically requires pre-processing (cell wall disruption) to improve human digestibility and absorption. This production difference makes large-scale cultivation of Spirulina simpler and more cost-effective, partly explaining why much of the sports nutrition research over the past two decades has focused on Spirulina.

In terms of nutritional composition, Spirulina’s protein content can reach 60–70% of dry weight, rich in high-quality protein including essential amino acids, and provides abundant iron, B vitamins, and γ-linolenic acid. The unique phycocyanin in Spirulina is both a potent antioxidant and believed to be involved in promoting nitric oxide synthesis. Chlorella contains high levels of chlorophyll, lutein/zeaxanthin, β-carotene, polyunsaturated fatty acids, and soluble dietary fiber. These components give Chlorella functions beyond antioxidant effects, including lipid regulation, cholesterol binding, and potential NO-promoting mechanisms.

These differences lead to different emphases in their application in sports: Spirulina is often studied for both enhancing endurance and promoting recovery, while Chlorella research mostly focuses on improving aerobic endurance and cardiovascular health. For example, several trials using a protocol of 6 g/day of Chlorella for 4 weeks consistently observed significant increases in VO_2_max in young men. In contrast, Spirulina trial protocols vary in dose from 1.5 g to 7.5 g/day and duration from one week to two months, with results sometimes showing greater variability. Overall, Chlorella intervention studies consistently support aerobic capacity improvement, while Spirulina studies cover both endurance performance and antioxidant/anti-inflammatory markers, with results more influenced by subject populations and study design differences.

Differences in intervention implementation across studies may also affect result interpretation. First, regarding dosage and duration, there is no consensus on an optimal protocol. Doses for Spirulina in existing studies range widely from about 1.5 g/day up to 7.5 g/day, and durations range from acute single-dose supplementation to chronic supplementation lasting up to 8 weeks. Chlorella doses are relatively concentrated, often using 6 g/day for 3–4 weeks, likely based on protocols yielding positive results in existing literature. Differences in dose and duration directly impact the accumulation and efficacy of active algae components in the body. Interestingly, some preliminary studies suggest a single high dose of algae can enhance the body’s antioxidant status shortly after exercise, but most endurance and recovery benefits appear after several weeks of consistent supplementation. Future research should compare acute versus chronic supplementation effects.

Second is the issue of combining supplementation with training programs: some studies incorporate systematic training for subjects alongside algae supplementation to examine nutrient-training interactions. Evidence suggests algae supplementation may amplify positive adaptations from regular training. For example, one study reported that Spirulina combined with an endurance training program produced greater improvements in exercise capacity, including additional gains in peak power and mean power, compared to training alone. Researchers attributed this to the high biological value of protein and amino acids in Spirulina improving the muscle anabolic environment during training. This finding suggests that adding algae supplements to training populations may yield better adaptive effects than training alone, but specific mechanisms require further investigation.

Furthermore, macroalgae preparations may differ from microalgae in functional focus. Research on macroalgae extracts for sports nutrition is relatively scarce, but some results are emerging. For instance, fucoidan from brown algae has garnered attention for its anti-inflammatory and immunomodulatory properties and has been trialed for alleviating inflammatory stress from repeated high-intensity exercise. A randomized crossover trial published in the International Journal of Sport Nutrition and Exercise Metabolism evaluated the effect of fucoidan on immune-inflammatory responses after high-intensity interval exercise. It found that, compared to placebo, the supplementation group showed a slight increase in the anti-inflammatory cytokine IL-10 30 min post-exercise, with no adverse effects. However, fucoidan did not show significant effects on performance indicators including peak and mean power. This suggests that, in the short term, this macroalgae extract primarily affects immune-inflammatory pathways, with limited direct impact on enhancing performance. It also hints that macroalgae supplements may be more suitable for promoting recovery and health management rather than typical acute performance enhancement. It should be noted that macroalgae are rich in iodine and unique polyphenols which may benefit metabolism and antioxidant defense, but excessive iodine intake poses potential risks. Therefore, different algae preparations have distinct characteristics in composition and function. Sports nutrition intervention strategies should select appropriate algae species and doses based on specific goals (enhancing endurance or accelerating recovery) and population characteristics. Simultaneously, strengthening direct comparative studies of various algae in exercise contexts will help clarify their respective advantages and optimal application strategies.

### 4.5. Insights from Subgroup Analyses: Moderating Factors and Heterogeneity

To further dissect the substantial between-study heterogeneity observed in our primary outcomes, pre-planned subgroup analyses (Figure 6) revealed several critical moderating factors.

First, regarding exercise modality, the ergogenic benefits of algae supplementation appeared more pronounced in aerobic-focused tasks (SMD = 0.79) compared to mixed-exercise protocols (SMD = 0.36). Conversely, the attenuation of muscle damage (CK/LDH) was most substantial following resistance exercise (SMD = −1.04). This divergence suggests a task-specific efficacy: algae-derived circulatory and metabolic enhancements primarily benefit continuous oxidative tasks, whereas their potent anti-inflammatory properties are most effectively recruited under conditions of high mechanical tension and structural muscle damage (e.g., eccentric resistance loading).

Second, the specific algae taxa notably influenced the outcomes. For performance metrics, Chlorella yielded the largest pooled effect (SMD = 1.39), which may be attributed to its unique nutrient matrix that heavily promotes nitric oxide synthesis and cardiovascular compliance. In contrast, for recovery outcomes, although Spirulina showed a stable protective effect (SMD = −0.39), specific extracts like seaweed-derived polysaccharides (SMD = −1.38) and *Haematococcus pluvialis*-derived astaxanthin (SMD = −2.53) displayed massive effect sizes.

However, a critical methodological caveat must be addressed regarding these specific recovery subgroups. The remarkably large effects observed for seaweed polysaccharides and astaxanthin were each driven by single studies (k = 1) in our sub-analysis. Consequently, these extreme SMD values must be interpreted with extreme caution. They should not be misconstrued as definitive proof of superiority over Spirulina or Chlorella, but rather viewed as strong preliminary signals that warrant targeted, large-scale investigation in future RCTs.

Finally, analyzing the participant populations revealed that the general, recreationally active population experienced slightly greater benefits in both performance (SMD = 0.70) and recovery (SMD = −0.97) compared to competitive athletes (SMD = 0.61 and −0.27, respectively). This is physiologically consistent with the “ceiling effect” often observed in elite athletes, whose highly developed endogenous antioxidant networks and optimized baseline physiological states render them less responsive to exogenous nutritional interventions than their untrained counterparts.

### 4.6. Study Limitations and Future Directions

Although the present meta-analysis suggests that algae supplementation may be beneficial for certain performance and recovery outcomes, the findings should be interpreted with caution in light of several important limitations.

First, the term “algae supplementation” encompasses a wide range of biologically distinct products, including Spirulina, Chlorella, brown algal extracts, and astaxanthin. Pooling these interventions provides a broad overview of algae-derived supplements but inevitably masks differences in composition, mechanisms, and efficacy. Although subgroup analyses by algae type were conducted, the small number of studies within each subgroup limits the strength of any comparative conclusions. As a result, the pooled estimates should be viewed as descriptive summaries of a heterogeneous literature rather than evidence supporting any specific algae product.

Second, most included studies involved relatively small samples and were conducted primarily in young, male, trained participants. This narrow population focus limits the generalizability of the findings to other groups, such as female athletes, older adults, and recreationally active individuals, who may respond differently to supplementation.

Third, substantial variability in study design—including exercise modality, outcome selection, supplementation dose, duration, and timing—contributed to inconsistent findings and prevented the identification of clear dose–response relationships. This heterogeneity reduces the precision of pooled estimates and complicates practical interpretation.

Fourth, aggregating results across heterogeneous outcomes carries an inherent risk of overinterpretation. We sought to more clearly separate outcomes showing no clear effect, outcomes with small but statistically significant effects of uncertain practical relevance (e.g., WRmax), and outcomes with potentially meaningful but variable effects (e.g., TTE and CK). This distinction underscores that statistically significant pooled effects should not be interpreted as evidence of uniform or robust efficacy.

Fifth, although our search strategy identified zero eligible non-English or non-Chinese publications during the screening phase, we acknowledge that our selected databases predominantly index English and Chinese literature. This inherent database bias may limit the capture of relevant trials published in other regional languages. Therefore, we must clearly state that language bias cannot be completely excluded from this meta-analysis. However, given the robustness of our results and supporting evidence regarding the limited impact of language restrictions [23], we believe our overall conclusions remain statistically stable.

Finally, although formal assessments did not consistently indicate publication bias, small-study effects cannot be fully excluded, particularly for recovery-related biomarkers where the number of available studies was limited.

Taken together, these limitations highlight several priorities for future research. Larger, well-powered randomized controlled trials are needed, with more diverse participant samples that include women, older adults, and athletes from different sporting backgrounds. Greater standardization of supplementation protocols—including dose, duration, and timing—would also improve comparability across studies and help clarify dose–response relationships.

In addition, more mechanistic research is warranted to better understand how algae-derived supplements influence exercise performance and recovery. Integrating mechanistic biomarkers into human trials, alongside targeted investigations of specific bioactive components, may help identify the conditions under which supplementation is most likely to be effective. Finally, greater attention should be given to product quality and processing methods, as differences in manufacturing may substantially influence bioavailability and efficacy.

Overall, algae supplementation remains a promising but still developing area of sports nutrition research. Clearer mechanistic insight and more consistent study designs will be essential to define its practical value and appropriate applications.

## 5. Conclusions

This systematic review and meta-analysis indicates that seaweed and microalgae supplementation may provide selective and context-dependent benefits for exercise performance and post-exercise recovery, rather than uniform efficacy across outcomes.

For aerobic exercise capacity, pooled results showed inconsistent but directionally favorable effects, with highly variable improvements in time to exhaustion and a borderline effect on VO_2_max. Regarding post-exercise recovery, algae supplementation was associated with a moderate attenuation of creatine kinase, although this finding was characterized by substantial heterogeneity and limited consistency across studies, while evidence for other recovery-related markers remained inconclusive.

Marked heterogeneity in algae type, supplementation protocols, participant characteristics, and exercise modalities complicates interpretation and limits generalizability. Taken together, algae supplementation may serve as a complementary sports nutrition strategy under specific conditions, particularly in endurance or high-intensity exercise contexts, but its practical value depends on multiple contextual factors.

Future research should focus on larger, standardized randomized trials, including direct comparisons between algae types, to clarify mechanisms, optimize intervention strategies, and define population-specific applications.

## Figures and Tables

**Table 1:** Characteristics of the included studies.

Author (Year)	Country/Region	Population	Sample Size (Intervention/Control)	Age	Gender (% Male)	Exercise Type	Intervention (Dose/Duration)	Physical Performance (TTE, VO_2_max)	Recovery (e.g., CK, LDH, CRP, IL-6, RPE, DOMS)	Formulation	Result
Bloomer (2005) [31]	USA	Resistance-trained healthy adults	10/10	25.1 ± 1.6	100.0%	Eccentric knee extensions (10 × 10 85% eccentric 1-RM)	BioAstin (*Haematococcus pluvialis* extract) ≈ 4 mg astaxanthin/day; 3 weeks pre + 4 days post	1-RM concentric strength (NS)	CK(NS); muscle soreness (VAS)(NS)	Capsules	No benefit on soreness, CK, 1-RM, or MIF; MDF lower vs. placebo at 10–72 h
Lu (2006) [32]	Taiwan, China	Healthy college students	8/8	20.7 ± 0.8	37.5%	Bruce incremental treadmill to exhaustion	Spirulina 7.5 g/day, 3 weeks	TTE ↑	MDA ↓; LDH show significant time × treatment interaction; CK: no significant change	Capsules	Spirulina improved antioxidant profile and extended TTE within group; mixed effects on damage markers; between-group ANCOVA favored Spirulina for GPx/LDH; CK non-significant.
Kalafati (2010) [33]	Greece	Healthy moderately trained men	9 (cross-over)	23.3 ± 1.7	100.0%	2 h treadmill run 70–75% VO_2_max + 95% VO_2_max to exhaustion	Spirulina 6 g/day for 4 weeks	TTE ↑ (*p* = 0.048); VO_2_max unchanged	GSH ↑ at rest & 24 h post; TBARS ↑ only in placebo; CK ↑ after exercise both groups;	Capsules	Spirulina improved endurance and fat oxidation; attenuated lipid peroxidation; no change in CK or TAC
Oh (2010) [34]	South Korea	Healthy male college students	20 (cross-over)	20.9 ± 2.0	100.0%	30 min submax treadmill + incremental to exhaustion	40 mg ECP (Ecklonia cava polyphenol) in 100 mL drink; acute pre-exercise	VO_2_max (NS); TTE ↑ (*p* < 0.05)	Lactate ↓ (–8.3%, NS)	Functional drink	ECP increased endurance time and glucose maintenance with a trend toward lower lactate; mechanisms speculative (antioxidant & vasodilatory effects).
Umemoto & Otsuki (2014) [35]	Japan	Healthy young men & women	10 (cross-over)	21.3 ± 0.3	70.0%	Incremental cycling to exhaustion	Chlorella 30 tablets/day (15 × 2) for 4 weeks	VO_2_peak ↑ (37.9 → 41.4 mL/kg/min, *p* = 0.003); VO_2_max ↑ (+4.3 mL/kg/min, *p* = 0.006); WRmax ↑ (+18 W, *p* = 0.05)	HRpeak,	Tablets	Chlorella significantly improved VO_2_peak and VO_2_max, suggesting enhanced aerobic capacity likely via multi-nutrient synergy.
Zempo-Miyaki (2017) [36]	Japan	Healthy young men with insufficient micronutrient intake	17/17	19.6–20.1	100.0%	Incremental cycling to exhaustion	Chlorella 30 tablets/day (15 × 2) for 4 weeks	VO_2_max ↑ (42.1 → 44.9 mL/kg/min, *p* < 0.05)	NR	Tablets	4-week Chlorella intake improved VO_2_max but not B_2_ levels; nutrient sufficiency of B_2_ rose from 67%→86%.
Chidley & Davison (2018) [37]	UK	Healthy trained adults	13/13	29.1 ± 8.7	77.0%	2-day intensified cycling: VO_2_max test + HIIT + 90 min steady ride	Chlorella 6 g/day for 6 weeks (30 tabs/day, Sun Chlorella A)	VO_2_max used for intensity calibration (no change reported)	NR	Tablets	Chlorella enhanced mucosal immunity (↑ sIgA secretion) but did not reduce URTI episodes.
Reid (2018) [38]	Korea	Healthy elderly adults	32/28	73.4 ± 5.5	NP	No structured exercise (habitual activity only)	Fermented Laminaria japonica (1.5 g/day) for 6 weeks	NR	TBARS,	Capsules	FST improved short-term memory, antioxidant enzyme activity, and functional mobility in the elderly.
Juszkiewicz (2019) [39]	Poland	Elite rowers (Polish National Team)	10/9	20.2 ± 0.8	100.0%	2000 m rowing ergometer test	Spirulina 1500 mg/day for 6 weeks	No change in power output or rowing time; LAmax ↓ slightly (NS)	IL-10 ↓	Capsules	Spirulina prevented Th2 shift and maintained Th1/Th2 cytokine balance during intensive training.
Gurney & Spendiff (2020) [40]	UK	Healthy untrained men	11 (crossover)	21.0 ± 1.0	100.0%	30-min arm crank at 55% VO_2_max + incremental to fatigue	Spirulina 6 g/day for 7 days	VO_2_ ↓ (−6%) *p* = 0.03; HR ↓ (−5 bpm) *p* = 0.022; VO_2_ at fatigue ↑ (+8.9%) *p* = 0.024; TTE NS	Hb ↑ (+7.9%, *p* = 0.005); no biochemical data reported	Capsules	Spirulina reduced submax VO_2_ & HR, raised Hb and VO_2_ at fatigue without affecting RER or TTE.
Chaouachi (2021) [41]	France	Elite male rugby players	11/11	25.8 ± 3.4	100.0%	Strength, vertical jump (SJ/CMJ), 10 m & 30 m sprint, Yo-Yo IRT-1	Spirulina 5.7 g/day × 7 weeks	Sprint and jump performance (NS)	No change in leg strength, both groups, no aerobic difference	Capsules	Small within-group gains in jump and sprint, no between-group effect on body composition or aerobic fitness.
Kashani (2022) [42]	Iran	Trained male taekwondo athletes	18 (cross-over)	26.6 ± 2.3	100.0%	2-h standardized taekwondo training (4 sessions)	Spirulina 8 g/day × 3 weeks	NP	↓ CK, ↓ LDH, ↓ IL-6 (*p* < 0.05); MDA NS	Powder drink	Spirulina enhanced antioxidant capacity, reduced inflammation and muscle damage without MDA change.
Chaouachi (2022) [43]	France	Elite rugby players	9/8	26.0 ± 3.8	100.0%	Yo-Yo Intermittent Recovery Test Level 2	Spirulina 5.7 g/day × 7 weeks	NP	↓ F2-Isop (–17.8% vs. ↑ in PLA), ↓ CRP (−27.5%), ↓ CK (−34.2%); LDH,	Capsules	Spirulina prevented lipid peroxidation, inflammation, and muscle damage; accelerated recovery within 24 h.
Cox (2023) [44]	Australia	Recreationally active males	12 (cross-over)	31.0 ± 9.6	100.0%	High-intensity cycling training (9 sessions/3 weeks)	Fucoidan (Undaria pinnatifida extract) 1 g/day × 3 weeks	↑ Mean & Peak Power (both groups); no between-group difference	NR	Capsules	Safe and tolerable; modest IgA response enhancement, no effect on performance or fecal markers.
McFadden (2023) [45]	USA	Healthy active adults	16 (cross-over)	21.2 ± 2.8	50.0%	Wingate anaerobic test × 9 (HIE)	Fucoidan 1 g/day × 2 weeks	No change in Peak or Mean Power	↑ IL-6 (*p* = 0.002), ↑ IL-10 (*p* = 0.005),	Capsules	Fucoidan enhanced IL-6/IL-10 recovery response; safe and tolerable, no ergogenic effect.
Ali (2024) [46]	UK	Recreationally active cyclists	17 (crossover)	23.0 ± 5.0	82.0%	20 min cycling 40% WRmax + VO_2_max test	Spirulina 6 g/day × 14 days	No change in VO_2_max, WRmax, TTF (*p* > 0.05)	↑ Hb (+3.4%, *p* = 0.047); No change in lactate, HR	Capsules	Hb increased, no ergogenic effect in submaximal/maximal cycling.
Gurney (2024) [47]	UK	Trained male cyclists	14 (cross-over)	37.0 ± 8.0	100.0%	Submax cycling (55% Wmax), 16.1 km TT, 3 × 20 s RSPT	Chlorella 6 g/day × 21 days	↑ Peak Power (+5.3%), ↑ Avg Power (+5.6%); TT NS	↓ Lactate (–12%), ↓ HR (–4%), ↑ Hb (+4.2%), VO_2_ NS	Capsules	Improved sprint power & submax homeostasis; no change in VO_2_max or TT.
Krokidas (2024) [48]	Greece	Healthy adults	14 (cross-over)	24.5 ± 3.8	57.0%	Eccentric elbow flexion	Spirulina Nigrita 42 mg/kg BW × 15 days	NR	CK ↑ (48–72 h male only), LDH ↑ (48–72 h male only), VAS ↑ (NS),	Capsules	No significant improvement; trend toward slightly worse ROM and CK.
La Mantia (2024) [49]	Italy	Male water polo players	10/10	25.0 ± 3.0	100.0%	Water polo training (5 sessions/week)	Spirulina extract 15 mL × 2/day × 8 weeks + Copper 22.5% NRV	NR	CK ↑ (186 → 289 U/L, *p* = 0.115, NS)	Liquid extract	Improved subjective performance; no objective biomarker improvement.
Hua (2018) [50]	China	Volleyball players	16/16	21.5 ± 2.1	50.00%	Single high-intensity resistance exercise	Chlorella powder 4.5 g/day × 35 d	NP	CK ↑ (*p* < 0.05), NO change in LDH	Tablets	Chlorella reduced DOMS after resistance exercise, but CK increased and LDH was unchanged.
Zhao (2016) [51]	China	Long-distance runners	20/20	22.5 ± 1.1	50.00%	Endurance running at 80% maximal exercise intensity after 30 min adaptive slow running	Algae peptide sports supplement 60 mL/day × 90 d (algal peptide 25.26%)	TTE ↑ (*p* < 0.05)	IL-6/IL-10 ↑ (*p* < 0.05)	Mixed drink	Algae peptide improved TTE and increased exercise-related cytokine responses, with no adverse events reported.
Zhang (2023) [52]	China	Football players	10/10	19.69 ± 0.92	100.00%	Ball training, strength, endurance, and tactical training, 6 d/week for 8 weeks	Spirulina extract 3 g/day × 56 d	WRmax ↑ (*p* < 0.05) average; power ↑ (*p <* 0.05)	mean hemoglobin concentration ↑	Tablets	Spirulina improved power-related performance, with broader gains observed in the supplementation group.

1-RM, one-repetition maximum; HIE, high-intensity exercise; TTE, time to exhaustion; TT, time trial; Yo-Yo IRT-1,Yo-Yo Intermittent Recovery Test Level 1; VO_2_max, maximal oxygen uptake; HR, heart rate; RPE, rating of perceived exertion; DOMS, delayed onset muscle soreness; CK, creatine kinase; LDH, lactate dehydrogenase; CRP, C-reactive protein; IL-6, interleukin-6; MDA, malondialdehyde; GPx, glutathione peroxidase; TAC, total antioxidant capacity; GSH, glutathione; TBARS, thiobarbituric acid reactive substances; VO_2_peak, peak oxygen uptake; MIF, maximal isometric force; MDF, median frequency; NP, Not Reported; NS, Not Significant. ↑ indicates a significant increase; ↓ indicates a significant decrease.

## Data Availability

All data generated or analyzed during this study are included in this published article and its Supplementary Materials. The datasets used or analyzed during the current study are available from the corresponding author upon reasonable request.

## Supplementary Materials

The following supporting information can be downloaded at: https://www.mdpi.com/article/10.3390/nu18081289/s1. Section S1 presents the complete search strategies used in PubMed, Cochrane Library, Embase, Web of Science, EBSCOhost, and CNKI. Section S2 provides the risk-of-bias summary (Figure S1) for all included randomized controlled trials, evaluated using the RoB 2.0 tool. Section S3 displays the forest plot (Figure S2) examining the effects of algae supplementation on time-trial (TT) performance. Section S4 includes the forest plot (Figure S3) assessing lactate dehydrogenase (LDH) outcomes. Section S5 shows the pooled forest plot (Figure S4) for lipid peroxidation markers (MDA + TBARS). Section S6 presents the leave-one-out sensitivity analysis (Figure S5) evaluating the robustness of creatine kinase (CK) effect estimates. Section S7 contains the funnel plot (Figure S6) for CK, while Section S8 provides Egger’s regression test (Figure S7) assessing small-study effects for CK-related outcomes. Section S9 contains the full R code used for data preprocessing, effect-size computation, statistical modeling, and generation of all main-text and supplementary figures. Section S10 provides the completed PRISMA 2020 checklist documenting adherence to systematic review reporting standards.

## Abbreviations

VO_2_max	maximal oxygen uptake
TTE	time to exhaustion
WRmax	maximal workload/peak power output
TT	time trial
CK	creatine kinase
LDH	lactate dehydrogenase
CRP	C-reactive protein
IL-6	interleukin-6
TNF-α	tumor necrosis factor-alpha
MDA	malondialdehyde
SOD	superoxide dismutase
GPx	glutathione peroxidase
CAT	catalase
TAC	total antioxidant capacity
GSH	glutathione
TBARS	thiobarbituric acid reactive substances
AOPP	advanced oxidation protein products
ROS	reactive oxygen species
NO	nitric oxide
RCT	randomized controlled trial
PRISMA	Preferred Reporting Items for Systematic Reviews and Meta-Analyses
PROSPERO	International Prospective Register of Systematic Reviews
RoB 2	Risk of Bias 2 tool
SMD	standardized mean difference
CI	confidence interval
