# Supplementary material for: The Effects of Seaweed and Microalgae Supplementation on Exercise Performance and Recovery: A Systematic Review and Meta-Analysis
Source: Nutrients. 2026 Apr 19;18(8):1289. doi: 10.3390/nu18081289 (PMC13119196; doi:10.3390/nu18081289)
Supplement: Supplementary file 1 [file nutrients-18-01289-s001.zip › nutrients-4192456-supplementary.pdf]

**Supplementary materials**	**Page**
**Section S1.** Search strategy of PubMed, Cochrane Library, Embase, Web of Science, EBSCOhost,and CNKI.	2-3
**Section S2.** Figure S1. Risk of bias summary of the included randomized controlled trials.	4
**Section S3.**Figure S2. Forest plot of the effects of algae supplementation on time-trial (TT) performance.	5
**Section S4.** Figure S3. Forest plot of the effects of algae supplementation on lactate dehydrogenase (LDH).	6
**Section S5.** Figure S4. Forest plot of the effects of algae supplementation on lipid peroxidation (MDA + TBARS, pooled).	7
**Section S6.** Figure S5. Leave-one-out sensitivity analysis for the effects of algae supplementation on creatine kinase (CK).	8
**Section S7.** Figure S6. Funnel plot for creatine kinase (CK).	9
**Section S8.** Figure S7. Egger's regression test for small-study effects on creatine kinase (CK).	10
**Section S9.** R code for data processing, meta-analyses, and figure generation.	11-60
**Section S10.** PRISMA 2020 checklist	61-63

Section S1. Search strategy of PubMed, Cochrane Library, Embase, Web of Science, EBSCOhost, and CNKI

PubMed

(("Spirulina"[Mesh] OR "Chlorella"[Mesh] OR "Seaweed"[Mesh] OR spirulina OR arthrospira OR chlorella OR "ecklonia cava" OR fucoidan OR fucoxanthin OR "brown seaweed" OR "green algae" OR "microalgae" OR astaxanthin OR haematococcus)) AND("Exercise"[Mesh] OR "Physical Fitness"[Mesh] OR "Sports"[Mesh] OR exercise OR training OR sport OR athlete OR endurance OR aerobic OR anaerobic OR resistance)AND("Physical Endurance"[Mesh] OR "Exercise Tolerance"[Mesh] OR "Recovery of Function"[Mesh] OR "Muscle Fatigue"[Mesh] OR "Muscle Strength"[Mesh] OR "Biomarkers"[Mesh]OR performance OR recovery OR "VO2max" OR "time to exhaustion" OR "time trial" OR power OR fatigue OR soreness OR DOMS OR CK OR "creatine kinase" OR CRP OR "C-reactive protein" OR LDH OR inflammation) AND("Randomized Controlled Trial"[Publication Type] OR "Controlled Clinical Trial"[Publication Type] OR randomized OR randomised OR placebo OR crossover OR trial)

Web of Science

(spirulina OR arthrospira OR chlorella OR "ecklonia cava" OR fucoidan OR fucoxanthin OR "brown seaweed" OR "green algae" OR microalgae OR astaxanthin OR haematococcus)

AND (exercise OR training OR sport OR athlete OR endurance OR aerobic OR anaerobic OR resistance)AND (performance OR recovery OR "VO2max" OR "time to exhaustion" OR "time trial" OR power OR fatigue OR soreness OR DOMS OR CK OR "creatine kinase" OR CRP OR "C-reactive protein" OR LDH OR inflammation) AND (randomized OR randomised OR placebo OR crossover OR trial)

Embase

(spirulina OR chlorella OR seaweed OR spirulina OR arthrospira OR chlorella OR ecklonia cava OR fucoidan OR fucoxanthin OR brown seaweed OR green algae OR microalgae OR astaxanthin OR haematococcus) AND (physical activity OR exercise OR sport OR exercise OR training OR athlete OR endurance OR aerobic OR anaerobic OR resistance) AND (physical endurance OR exercise tolerance OR functional recovery OR muscle fatigue OR muscle strength OR biological marker OR performance OR recovery OR vo2max OR time to exhaustion OR time trial OR power OR fatigue OR soreness OR DOMS OR CK OR creatine kinase OR CRP OR c-reactive protein OR LDH OR inflammation)

EBSCO

(spirulina OR arthrospira OR chlorella OR "ecklonia cava" OR fucoidan OR fucoxanthin OR "brown seaweed" OR "green algae" OR microalgae OR astaxanthin OR haematococcus) AND (exercise OR training OR sport OR athlete OR endurance OR aerobic OR anaerobic OR "resistance training") AND (performance OR recovery OR "VO2max" OR "time to exhaustion" OR "time trial" OR power OR fatigue OR soreness OR DOMS OR CK OR "creatine kinase" OR CRP OR "C-reactive protein" OR LDH OR inflammation OR "oxidative stress") AND (randomized OR randomised OR placebo OR crossover OR "controlled trial" OR "clinical trial")

Cochrane Library

(spirulina OR arthrospira OR chlorella OR "ecklonia cava" OR fucoidan OR fucoxanthin OR "brown seaweed" OR "green algae" OR microalgae OR astaxanthin OR haematococcus)

AND(exercise OR training OR sport OR athlete OR endurance OR aerobic OR anaerobic OR "resistance training")AND(performance OR recovery OR "VO2max" OR "time to exhaustion" OR "time trial" OR

power OR fatigue OR soreness OR DOMS OR CK OR "creatine kinase" OR CRP OR "C-reactive protein" OR LDH OR inflammation OR "oxidative stress")

#1 "Spirulina" OR "Chlorella" OR "Seaweed" OR spirulina OR arthrospira OR chlorella OR "ecklonia cava" OR fucoidan OR fucoxanthin

#2 "brown seaweed" OR "green algae" OR "microalgae" OR astaxanthin OR haematococcus

#3 "Exercise" OR "Physical Fitness" OR "Sports" OR exercise OR training OR sport OR athlete OR endurance OR aerobic OR anaerobic OR resistance

#4 "Physical Endurance" OR "Exercise Tolerance" OR "Recovery of Function" OR "Muscle Fatigue" OR "Muscle Strength"

#5 recovery OR "VO2max" OR "time to exhaustion" OR "time trial" OR power OR fatigue OR soreness OR DOMS OR CK OR "creatine kinase" OR CRP OR "C-reactive protein" OR LDH OR inflammation

#6 (#1 OR #2) AND #3 AND (#4 OR #5)

CNKI

(螺旋藻 + 小球藻 + 海藻 + 褐藻 + 绿藻 + 微藻 + 藻类 + 海藻多糖 + 岩藻多糖 + 岩藻黄质 + 虾青素 + 雨生红球藻 + spirulina + chlorella + seaweed + "brown seaweed" + "green algae" + microalgae + astaxanthin + haematococcus)*(运动 + 体育运动 + 锻炼 + 训练 + 运动员 + 耐力 + 有氧 + 无氧 + 抗阻 + resistance + exercise + training + sport + athlete + endurance + aerobic + anaerobic

Section S2. Figure S1. Risk of bias summary of the included randomized controlled trials.

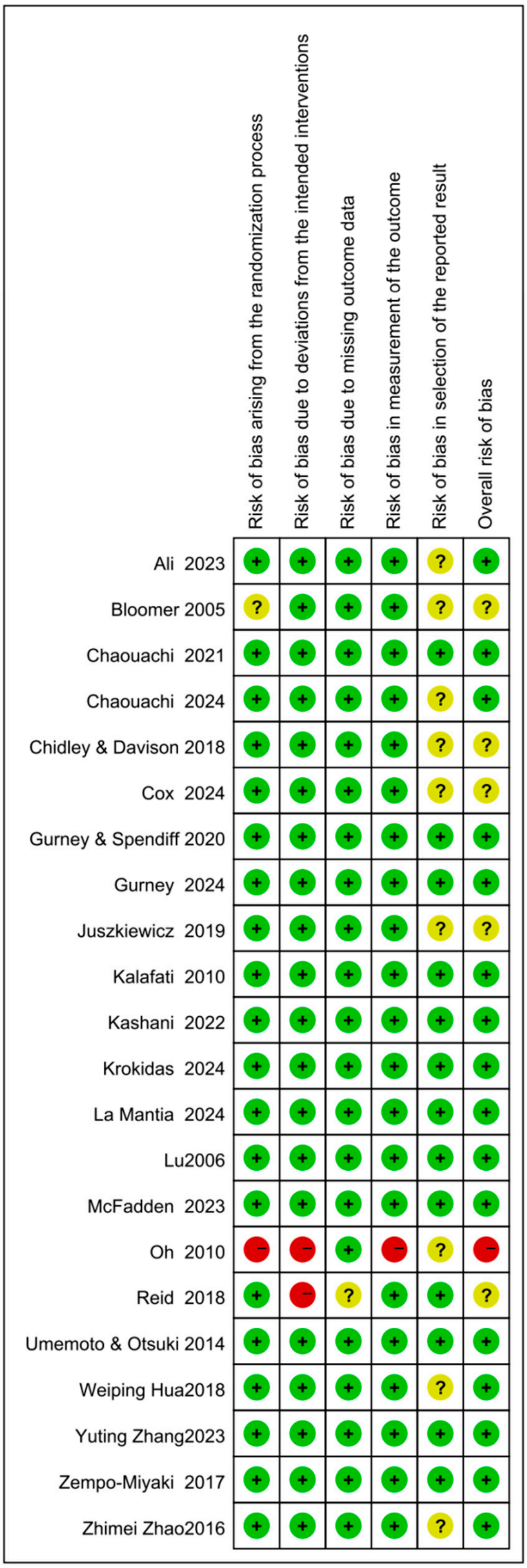

Figure S1. Risk-of-bias assessment of randomized controlled trials investigating algae or microalgae supplementation. The traffic-light plot summarizes the risk of bias for each domain of the RoB 2.0 tool (randomization process, deviations from intended interventions, missing outcome data, measurement of the outcome, selection of the reported result) and the overall risk of bias. Green, yellow, and red circles indicate low risk, some concerns, and high risk of bias, respectively.

Section S3. Figure S2. Forest plot of the effects of algae supplementation on time-trial (TT) performance.

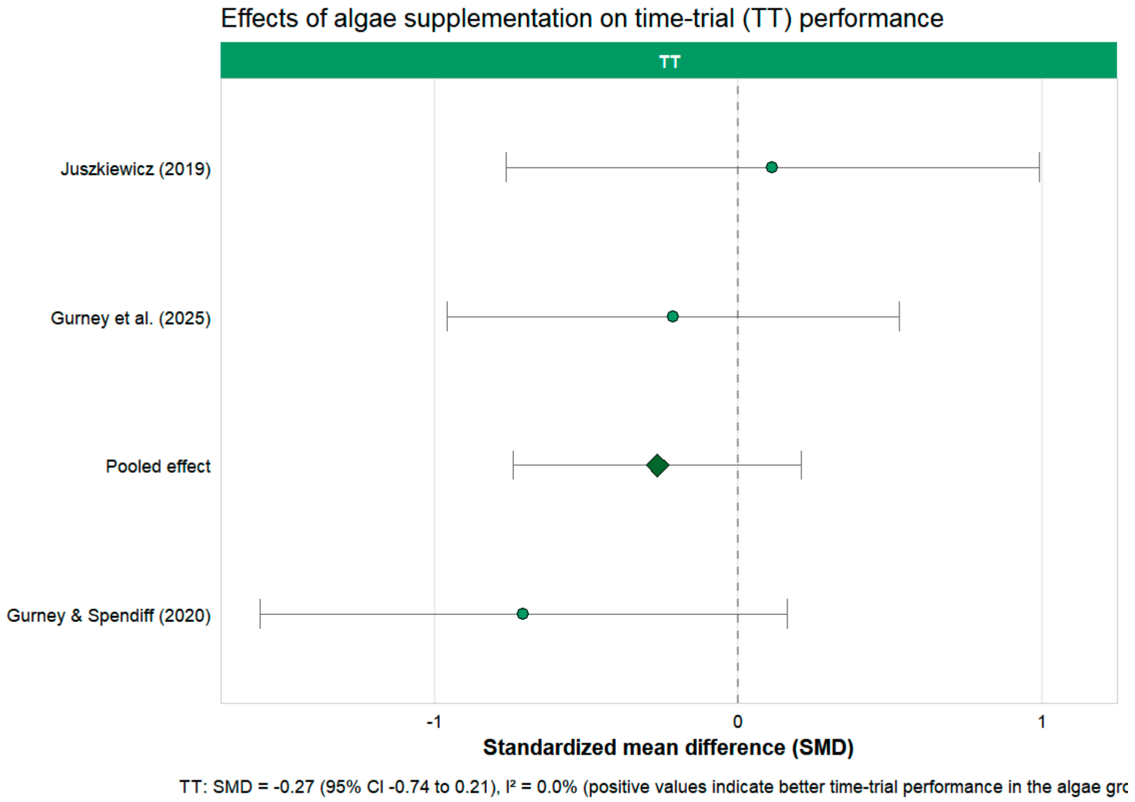

Figure S2. Effects of algae supplementation on time-trial (TT) performance.
Random-effects meta-analysis of standardized mean difference (SMD) comparing algae supplementation with control for TT performance. Positive SMD values indicate better TT performance in the algae group. The pooled effect was SMD = –0.27 (95% CI –0.74 to 0.21), I^2 = 0.0%, suggesting no statistically significant improvement in TT performance.

Section S4. Figure S3. Forest plot of the effects of algae supplementation on lactate dehydrogenase (LDH).

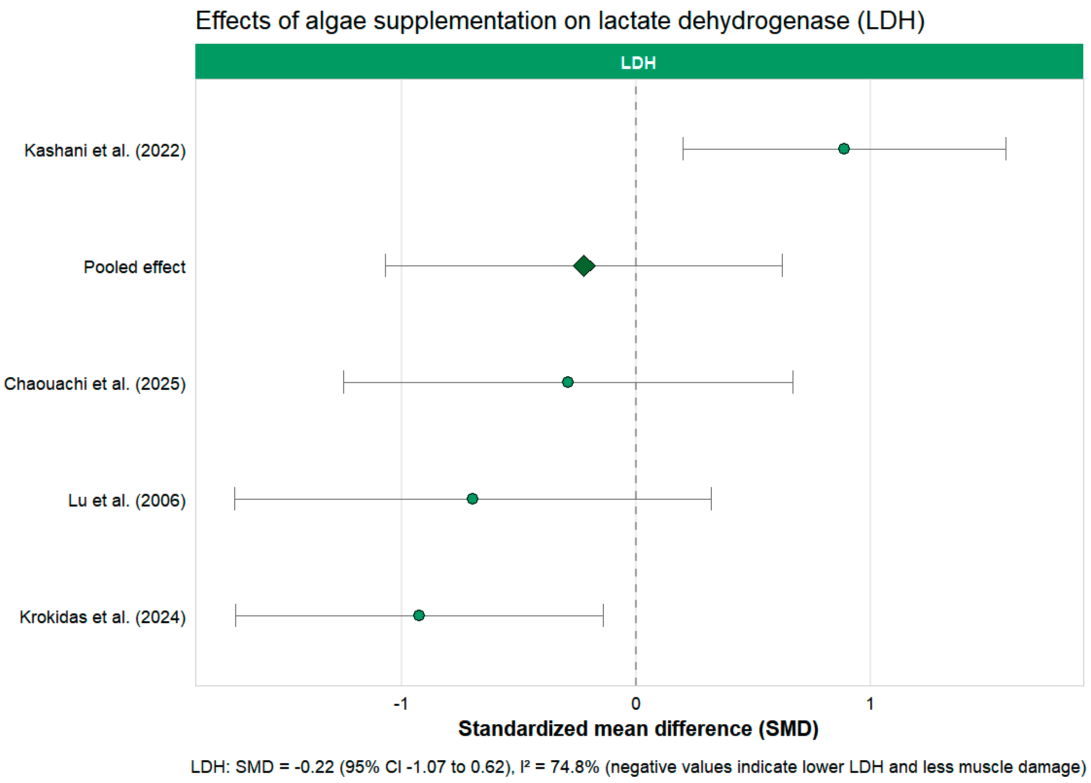

Figure S3. Effects of algae supplementation on lactate dehydrogenase (LDH).
Random-effects meta-analysis of SMD for post-exercise LDH. Negative SMD values indicate lower LDH levels and less muscle damage in the algae group. The pooled SMD was –0.22 (95% CI –1.07 to 0.62), with substantial heterogeneity (I^2 = 74.8%), indicating inconclusive evidence for a consistent LDH-lowering effect.

Section S5. Figure S4. Forest plot of the effects of algae supplementation on lipid peroxidation (MDA + TBARS, pooled).

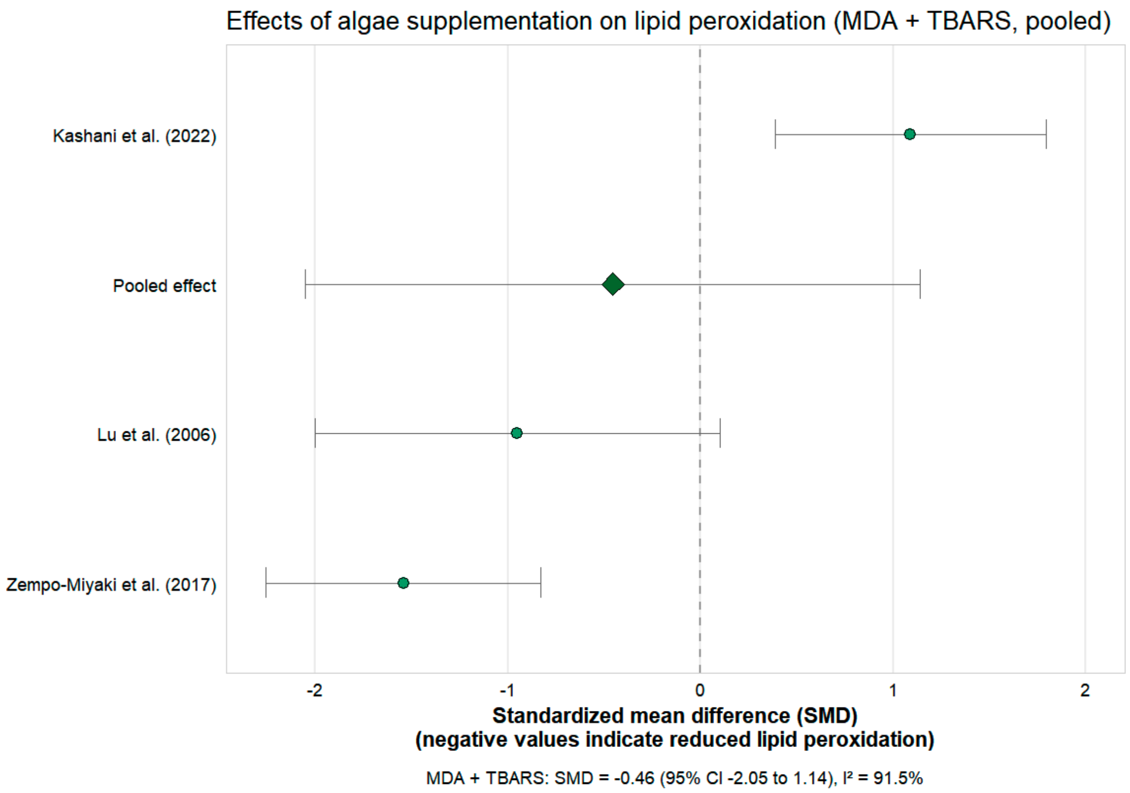

Figure S4. Effects of algae supplementation on lipid peroxidation (MDA + TBARS, pooled).
Random-effects meta-analysis of SMD for lipid peroxidation markers (MDA and TBARS). Negative SMD values indicate reduced lipid peroxidation in the algae group. The pooled SMD was –0.46 (95% CI –2.05 to 1.14), with very high heterogeneity (I^2 = 91.5%), suggesting inconsistent effects across studies.

Section S6. Figure S5. Leave-one-out sensitivity analysis for the effects of algae supplementation on creatine kinase (CK).

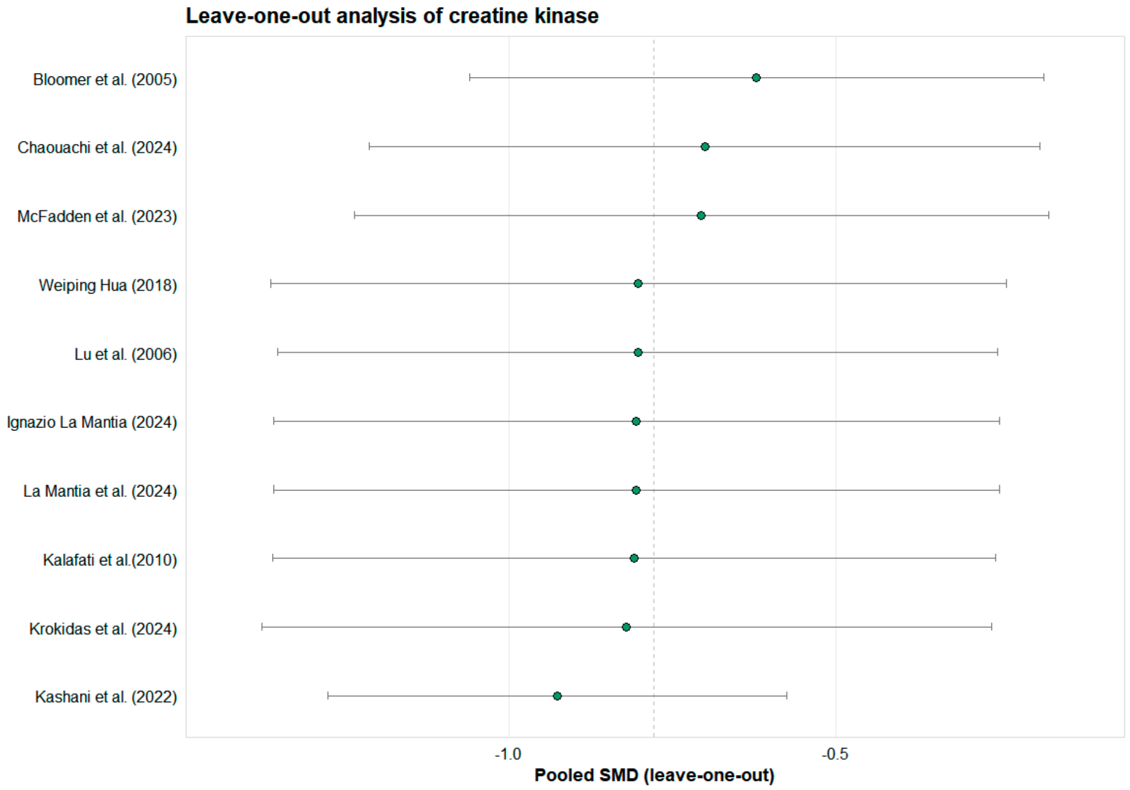

Figure S5. Leave-one-out sensitivity analysis for the effects of algae supplementation on creatine kinase (CK).
Each point represents the pooled SMD for CK after omitting one study at a time from the meta-analysis. The direction of effect consistently favors algae supplementation (lower CK levels), and the magnitude of the pooled SMD changes only slightly, indicating reasonable robustness of the overall findings.

Section S7. Figure S6. Funnel plot for creatine kinase (CK).

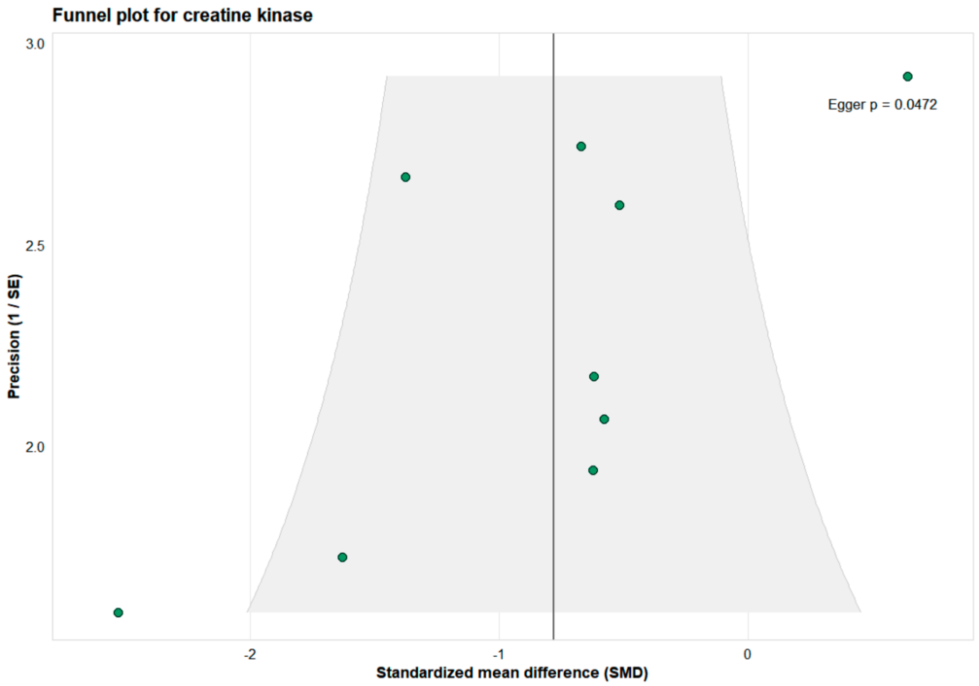

Figure S6. Funnel plot for creatine kinase (CK).
Funnel plot of SMD versus precision (1/SE) for CK, with the pooled random-effects estimate and contour-enhanced region. The distribution of points appears mildly asymmetric. Egger's regression test yielded p = 0.0472, indicating potential small-study effects or publication bias for CK outcomes.

Section S8. Figure S7. Egger's regression test for small-study effects on creatine kinase (CK)

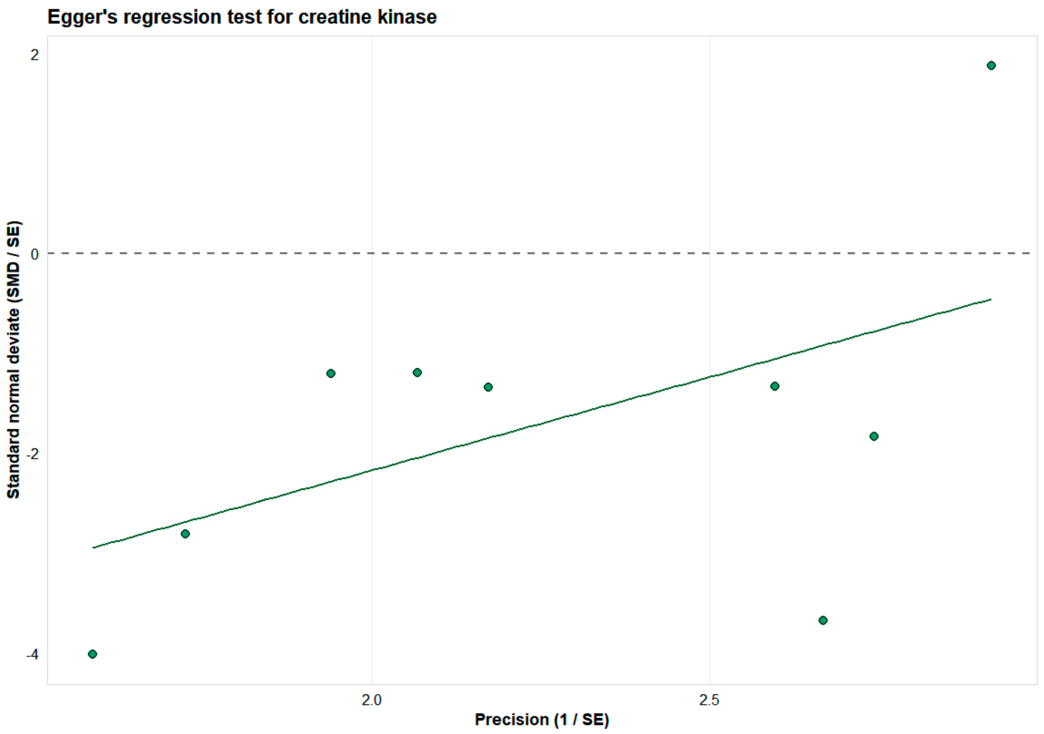

Figure S7. Egger's regression test for small-study effects on creatine kinase (CK).
Scatter plot of the standard normal deviate (SMD/SE) against precision (1/SE), with the fitted regression line from Egger's test. The intercept is significantly different from zero (p = 0.0472), supporting the presence of potential small-study effects or publication bias in CK outcomes.

Section S9. R code for data processing, meta-analyses, and figure generation

The following R code was used to preprocess the data, compute effect sizes, perform meta-analyses, and generate all figures presented in the main text and supplementary materials.

The code is organized by figure/topic for reproducibility. Users should adapt the working directory and file paths (e.g., dataextra.csv) to their local environment.

Section S9. R code for data processing, meta-analyses,and figure generation

```
###############################################################
## --------------------------------------------------------
## Section A. Packages and global plotting theme
```

```r
## --------------------------------------------------------
# Uncomment the following lines if the packages are not installed
# install.packages("DiagrammeR")
# install.packages("DiagrammeRsvg")
# install.packages("rsvg")
# install.packages("patchwork")
library(DiagrammeR)
library(DiagrammeRsvg)
library(rsvg)
library(readr)
library(dplyr)
library(stringr)
library(forcats)
library(ggplot2)
library(metafor)
library(patchwork)
## Nutrients-like ggplot2 theme (clean, minimal)
theme_nutrients <- function(base_size = 11) {
  theme_minimal(base_size = base_size) +
    theme(
      panel.grid.major.y = element_blank(),
      panel.grid.minor   = element_blank(),
      panel.border       = element_rect(colour = "grey80", fill = NA, linewidth = 0.4),
      axis.title         = element_text(face = "bold"),
      axis.text          = element_text(colour = "black"),
      strip.background   = element_rect(fill = "#009966", colour = NA),
```

```
    strip.text        = element_text(colour = "white", face = "bold"),
    legend.position   = "bottom"
  )
}
## --------------------------------------------------------
## Section B. Data import, cleaning, and helper functions
## --------------------------------------------------------
## Set working directory and read the main dataset
## (adapt the path to your local machine)
# setwd("C:/Users/NAME/Desktop")
dat_raw <- read_csv(
  "dataextra.csv",
  locale = locale(encoding = "GB18030"),
  show_col_types = FALSE
)

## Basic cleaning and harmonization
dat <- dat_raw %>%
  mutate(
    Indicator    = str_trim(Indicator),
    Study        = str_trim(Study),
    exercise_cat = str_trim(exercise_cat),
    dose_cat     = str_trim(dose_cat),
    duration_cat = str_trim(duration_cat),
    Fomulation   = str_trim(Fomulation),
    algae_cat    = str_trim(algae_cat)
```

```
  ) %>%
  mutate(
    across(
      c(`ΔMean_exp`, `ΔSD_exp`, `ΔMean_con`, `ΔSD_con`),
      ~ as.numeric(str_replace_all(as.character(.), "–", "-"))
    )
  )
## Helper function 1: run random-effects meta-analysis for one group
run_meta_for_group <- function(df_group, group_label = NULL) {
  esc <- escalc(
    measure = "SMD",
    m1i = `ΔMean_exp`, sd1i = `ΔSD_exp`, n1i = n_exp,
    m2i = `ΔMean_con`, sd2i = `ΔSD_con`, n2i = n_con,
    data  = df_group,
    vtype = "UB"
  )
  fit <- rma(yi, vi, data = esc, method = "REML")
  stud_df <- esc %>%
    mutate(
      type   = "Study",
      sei    = sqrt(vi),
      ci_lb  = yi - 1.96 * sei,
      ci_ub  = yi + 1.96 * sei,
      pooled = FALSE
    ) %>%
    select(Study, Indicator, yi, ci_lb, ci_ub, sei, type, pooled)
```

```
  pool_df <- tibble(
    Study     = "Pooled effect",
    Indicator = unique(df_group$Indicator)[1],
    yi        = as.numeric(fit$b[1]),
    ci_lb     = fit$ci.lb,
    ci_ub     = fit$ci.ub,
    sei       = fit$se,
    type      = "Pooled",
    pooled    = TRUE
  )

  out <- bind_rows(stud_df, pool_df) %>%
    mutate(group_label = group_label)

  list(fit = fit, df = out)
}
## Helper function 2: generic ggplot2 forest plot
plot_forest_gg <- function(df_forest,
                           xlab = "Standardized mean difference (SMD)",
                           vline_at = 0,
                           facet_var = NULL,
                           title = NULL,
                           caption = NULL) {

  df_forest <- df_forest %>%
    mutate(
```

```
    Study_plot = fct_reorder(Study, yi),
    is_pooled  = pooled
  )
p <- ggplot(df_forest, aes(x = yi, y = Study_plot)) +
  geom_vline(xintercept = vline_at,
             linetype = "dashed",
             colour = "grey60") +
  geom_errorbarh(
    aes(xmin = ci_lb, xmax = ci_ub),
    height   = 0.2,
    linewidth= 0.4,
    colour   = "grey40"
  ) +
  geom_point(
    data   = subset(df_forest, !is_pooled),
    shape  = 21,
    size   = 2.5,
    fill   = "#009966",
    colour = "black",
    stroke = 0.3
  ) +
  geom_point(
    data   = subset(df_forest, is_pooled),
    shape  = 23,
    size   = 4,
    fill   = "#006633",
```

```
    colour = "black",
    stroke = 0.4
  ) +
  scale_x_continuous(expand = expansion(mult = c(0.05, 0.1))) +
  labs(
    x       = xlab,
    y       = NULL,
    title   = title,
    caption = caption
  ) +
  theme_nutrients() +
  theme(
    plot.caption = element_text(
      hjust  = 0.5,
      size   = 9,
      margin = margin(t = 10)
    )
  )

if (!is.null(facet_var)) {
  p <- p + facet_wrap(vars(!!sym(facet_var)),
                      ncol = 1,
                      scales = "free_y")
}

p
```

```
}
## -------------------------------------------------------
## Section C. Figure 1 – PRISMA 2020 flow diagram
## -------------------------------------------------------
prisma_std <- grViz('
digraph prisma {

  graph [layout = dot,
         rankdir = TB,
         splines = ortho]
  # Global node style
  node [shape = box,
        style = "rounded",
        color = "black",
        fillcolor = "white",
        fontname = "Helvetica",
        fontsize = 10]
  edge [color = "black",
        arrowsize = 0.6]
  # Top title box
  top [label = "Identification of studies via databases and registers",
       shape = box,
       style = "rounded,filled",
       fillcolor = "#FFD966",
       fontsize = 11]
  # Main flow nodes
```

```
  id_db [label = "Records identified from databases (n = 1230)\\n\\nEmbase (n = 259)\\nPubMed (n = 209)\\nWeb of Science (n = 323)\\nCochrane Library (n = 107)\\nEBSCO (n = 143)\\nCNKI (n = 189)"]

  removed [label = "Records removed before screening:\\nDuplicate records removed (n = 265)"]

  screened [label = "Records screened (n = 965)"]

  excl_screen [label = "Records excluded (n = 845)"]

  elig [label = "Reports assessed for eligibility\\n(full-text) (n = 120)"]

  excl_reason [label = "Records excluded based on title and abstract screening:\\nReviews (n = 13)\\nSystematic reviews and meta-analyses (n = 10)\\nIneligible study design (n = 19)\\nIneligible population (n = 9)\\nIneligible intervention (n = 20)\\nIneligible outcome (n = 25)\\nHigh risk of bias (n = 2)"]

  included [label = "Studies included in systematic review (n = 22)"]

  meta_perf [label = "Studies included in the\\nquantitative synthesis\\n(Physical performance) (n = 14)"]

  meta_rec  [label = "Studies included in the\\nquantitative synthesis\\n(Recovery) (n = 11)"]

  # Stage labels

  node [shape = box,

        style = "filled",

        fillcolor = "#D9E1F2",

        fontsize = 10]

  lab_id   [label = "Identification"]

  lab_scr  [label = "Screening"]

  lab_elig [label = "Eligibility"]

  lab_incl [label = "Included"]

  # Edges

  top -> id_db

  id_db -> removed

  id_db -> screened

  screened -> excl_screen
```

```
  screened -> elig
  elig -> excl_reason
  elig -> included
  included -> meta_perf
  included -> meta_rec
  # Rank alignment
  {rank = same; id_db; removed}
  {rank = same; screened; excl_screen}
  {rank = same; elig; excl_reason}
  {rank = same; meta_perf; meta_rec}
  {rank = same; lab_id;   id_db}
  {rank = same; lab_scr;  screened}
  {rank = same; lab_elig; elig}
  {rank = same; lab_incl; included}
  lab_id   -> id_db    [style = "invis"]
  lab_scr  -> screened [style = "invis"]
  lab_elig -> elig     [style = "invis"]
  lab_incl -> included [style = "invis"]
}
')
# View in RStudio Viewer
prisma_std
# To export: export via DiagrammeRsvg + rsvg if needed.
## ---------------------------------------------------------
## Section D. Figure 2 – Forest plots for VO2max, TTE, WRmax
## ---------------------------------------------------------
```

```r
vo2_patterns <- c("VO2max", "VO₂max")
tte_patterns <- c("TTE", "Time to exhaustion", "time-to-exhaustion")
wr_patterns  <- c("WRmax", "Maximal work rate", "Peak power")
dat_perf <- dat %>%
  mutate(
    perf_group = case_when(
      str_detect(Indicator, str_c(vo2_patterns, collapse = "|")) ~ "VO2max",
      str_detect(Indicator, str_c(tte_patterns, collapse = "|")) ~ "TTE",
      str_detect(Indicator, str_c(wr_patterns,  collapse = "|")) ~ "WRmax",
      TRUE ~ NA_character_
    )
  ) %>%
  filter(
    !is.na(perf_group),
    !is.na(`ΔMean_exp`), !is.na(`ΔSD_exp`),
    !is.na(`ΔMean_con`), !is.na(`ΔSD_con`)
  )
perf_meta_list <- dat_perf %>%
  group_split(perf_group) %>%
  lapply(function(df_g) {
    gname <- unique(df_g$perf_group)
    run_meta_for_group(df_g, group_label = gname)
  })
perf_forest_df <- bind_rows(lapply(perf_meta_list, `[[`, "df")) %>%
  mutate(
    perf_group = factor(group_label,
```

```
                    levels = c("VO2max", "TTE", "WRmax")),
    Study = if_else(pooled, "Pooled effect", Study)
  )

## Summaries for caption
perf_stats <- lapply(perf_meta_list, function(x) {
  fit <- x$fit
  tibble(
    perf_group = x$df$group_label[1],
    SMD        = as.numeric(fit$b[1]),
    CI_lb      = fit$ci.lb,
    CI_ub      = fit$ci.ub,
    I2         = fit$I2,
    tau2       = fit$tau2,
    Q          = fit$QE,
    Q_p        = fit$QEp
  )
}) %>%
  bind_rows()
caption_text <- perf_stats %>%
  mutate(
    txt = sprintf(
      "%s: SMD = %.2f (95%% CI %.2f to %.2f), I² = %.1f%%",
      perf_group, SMD, CI_lb, CI_ub, I2
    )
  ) %>%
```

```
  pull(txt) %>%
  paste(collapse = "  |  ")


fig2 <- plot_forest_gg(
  df_forest = perf_forest_df,
  xlab      = "Standardized mean difference (SMD)",
  vline_at  = 0,
  facet_var = "perf_group",
  title     = "Effects of algae supplementation on aerobic performance outcomes",
  caption   = caption_text
)
fig2
# ggsave("Fig2_performance_forest.png", fig2, width = 7, height = 9, dpi = 600)
## -------------------------------------------------------
## Section E. Supplementary Figure S2 – TT forest plot
## -------------------------------------------------------
tt_patterns <- c("\\bTT\\b", "Time trial", "time trial", "time-trial")
dat_tt <- dat %>%
  filter(
    str_detect(Indicator, str_c(tt_patterns, collapse = "|")),
    !is.na(`ΔMean_exp`), !is.na(`ΔSD_exp`),
    !is.na(`ΔMean_con`), !is.na(`ΔSD_con`)
  )
tt_meta_res <- run_meta_for_group(dat_tt, group_label = "TT")


tt_forest_df <- tt_meta_res$df %>%
```

```
  mutate(
    Study = if_else(pooled, "Pooled effect", Study),
    panel = "TT"
  )
fit_tt <- tt_meta_res$fit
tt_stats <- tibble(
  marker = "TT",
  SMD    = as.numeric(fit_tt$b[1]),
  CI_lb  = fit_tt$ci.lb,
  CI_ub  = fit_tt$ci.ub,
  I2     = fit_tt$I2,
  tau2   = fit_tt$tau2,
  Q      = fit_tt$QE,
  Q_p    = fit_tt$QEp
)
caption_text_tt <- sprintf(
  "TT: SMD = %.2f (95%% CI %.2f to %.2f), I² = %.1f%% (positive values indicate better time-trial performance in the algae group)",
  tt_stats$SMD, tt_stats$CI_lb, tt_stats$CI_ub, tt_stats$I2
)
fig_tt <- plot_forest_gg(
  df_forest = tt_forest_df,
  xlab      = "Standardized mean difference (SMD)",
  vline_at  = 0,
  facet_var = "panel",
  title     = "Effects of algae supplementation on time-trial (TT) performance",
```

```
  caption   = caption_text_tt
)
Fig.S2
# ggsave("Supp_Fig_S2_TT_forest.png", fig_tt, width = 7, height = 7, dpi = 600)
## ---------------------------------------------------------
## Section F. Supplementary Figure S3 – LDH forest plot
## ---------------------------------------------------------


ldh_patterns <- c("LDH", "Lactate dehydrogenase")
dat_ldh <- dat %>%
  filter(
    str_detect(Indicator, str_c(ldh_patterns, collapse = "|")),
    !is.na(`ΔMean_exp`), !is.na(`ΔSD_exp`),
    !is.na(`ΔMean_con`), !is.na(`ΔSD_con`)
  )
ldh_meta_res <- run_meta_for_group(dat_ldh, group_label = "LDH")


ldh_forest_df <- ldh_meta_res$df %>%
  mutate(
    Study = if_else(pooled, "Pooled effect", Study),
    panel = "LDH"
  )
fit_ldh <- ldh_meta_res$fit
ldh_stats <- tibble(
  marker = "LDH",
  SMD    = as.numeric(fit_ldh$b[1]),
```

```
  CI_lb  = fit_ldh$ci.lb,
  CI_ub  = fit_ldh$ci.ub,
  I2     = fit_ldh$I2,
  tau2   = fit_ldh$tau2,
  Q      = fit_ldh$QE,
  Q_p    = fit_ldh$QEp
)
caption_text_ldh <- sprintf(
  "LDH: SMD = %.2f (95%% CI %.2f to %.2f), I² = %.1f%% (negative values indicate lower LDH and less muscle damage in the algae group)",
  ldh_stats$SMD, ldh_stats$CI_lb, ldh_stats$CI_ub, ldh_stats$I2
)
fig_ldh <- plot_forest_gg(
  df_forest = ldh_forest_df,
  xlab      = "Standardized mean difference (SMD)",
  vline_at  = 0,
  facet_var = "panel",
  title     = "Effects of algae supplementation on lactate dehydrogenase (LDH)",
  caption   = caption_text_ldh
)
fig_ldh
# ggsave("Supp_Fig_S3_LDH_forest.png", fig_ldh, width = 7, height = 7, dpi = 600)
## -------------------------------------------------------
## Section G. Supplementary Figure S4 – MDA + TBARS forest plot
## -------------------------------------------------------
mda_tbars_patterns <- c("MDA", "TBARS", "Thiobarbituric acid reactive substances")
```

```
dat_ox_mix <- dat %>%
  filter(
    str_detect(Indicator, str_c(mda_tbars_patterns, collapse = "|")),
    !is.na(`ΔMean_exp`), !is.na(`ΔSD_exp`),
    !is.na(`ΔMean_con`), !is.na(`ΔSD_con`)
  )


ox_mix_res <- run_meta_for_group(dat_ox_mix, group_label = "MDA/TBARS")


ox_mix_forest_df <- ox_mix_res$df %>%
  mutate(
    Study = if_else(pooled, "Pooled effect", Study)
  )
fit_ox <- ox_mix_res$fit
ox_mix_stats <- tibble(
  marker   = "MDA + TBARS (pooled)",
  SMD      = as.numeric(fit_ox$b[1]),
  CI_lb    = fit_ox$ci.lb,
  CI_ub    = fit_ox$ci.ub,
  I2       = fit_ox$I2,
  tau2     = fit_ox$tau2,
  Q        = fit_ox$QE,
  Q_p      = fit_ox$QEp
)
caption_text_ox_mix <- sprintf(
  "MDA + TBARS: SMD = %.2f (95%% CI %.2f to %.2f), I² = %.1f%%",
```

```
  ox_mix_stats$SMD, ox_mix_stats$CI_lb, ox_mix_stats$CI_ub, ox_mix_stats$I2
)
fig_mda <- plot_forest_gg(
  df_forest = ox_mix_forest_df,
  xlab      = "Standardized mean difference (SMD)\n(negative values indicate reduced lipid peroxidation)",
  vline_at  = 0,
  facet_var = NULL,
  title     = "Effects of algae supplementation on lipid peroxidation (MDA + TBARS, pooled)",
  caption   = caption_text_ox_mix
)
fig_mda
# ggsave("Supp_Fig_S4_MDA_TBARS_forest.png", fig_mda, width = 6.5, height = 6, dpi = 600)
## ----------------------------------------------------------
## Section H. CK forest plot (main text, if used)
## ----------------------------------------------------------
ck_patterns <- c("CK", "Creatine kinase")
dat_ck <- dat %>%
  filter(
    str_detect(Indicator, str_c(ck_patterns, collapse = "|")),
    !is.na(`ΔMean_exp`), !is.na(`ΔSD_exp`),
    !is.na(`ΔMean_con`), !is.na(`ΔSD_con`)
  )
ck_meta_res <- run_meta_for_group(dat_ck, group_label = "CK")
ck_forest_df <- ck_meta_res$df %>%
  mutate(
    Study = if_else(pooled, "Pooled effect", Study),
```

```
    panel = "CK"
  )
fit_ck <- ck_meta_res$fit
ck_stats <- tibble(
  marker = "CK",
  SMD    = as.numeric(fit_ck$b[1]),
  CI_lb  = fit_ck$ci.lb,
  CI_ub  = fit_ck$ci.ub,
  I2     = fit_ck$I2,
  tau2   = fit_ck$tau2,
  Q      = fit_ck$QE,
  Q_p    = fit_ck$QEp
)
caption_text_ck <- ck_stats %>%
  mutate(
    txt = sprintf(
      "CK: SMD = %.2f (95%% CI %.2f to %.2f), I² = %.1f%% (negative values indicate lower CK and less muscle damage in the algae group)",
      SMD, CI_lb, CI_ub, I2
    )
  ) %>%
  pull(txt)
fig_ck <- plot_forest_gg(
  df_forest = ck_forest_df,
  xlab      = "Standardized mean difference (SMD)",
  vline_at  = 0,
```

```
  facet_var = "panel",
  title     = "Effects of algae supplementation on creatine kinase (CK)",
  caption   = caption_text_ck
)
fig_ck
# ggsave("Fig_CK_forest.png", fig_ck, width = 7, height = 7, dpi = 600)
## ---------------------------------------------------------
## Section I. Rainforest plot summarizing pooled outcomes
## ---------------------------------------------------------
build_meta_for_outcome <- function(pattern_vec, label) {
  df_g <- dat %>%
    filter(
      str_detect(Indicator, str_c(pattern_vec, collapse = "|")),
      !is.na(`ΔMean_exp`), !is.na(`ΔSD_exp`),
      !is.na(`ΔMean_con`), !is.na(`ΔSD_con`)
    )

  if (nrow(df_g) == 0) return(NULL)

  res <- run_meta_for_group(df_g, group_label = label)
  fit <- res$fit

  tibble(
    outcome = label,
    SMD     = as.numeric(fit$b[1]),
    CI_lb   = fit$ci.lb,
```

```
    CI_ub   = fit$ci.ub,
    I2      = fit$I2
  )
}

rain_list <- list(
  build_meta_for_outcome(c("VO2max", "VO₂max"), "VO2max"),
  build_meta_for_outcome(c("TTE", "Time to exhaustion", "time-to-exhaustion"), "TTE"),
  build_meta_for_outcome(c("WRmax", "Maximal work rate", "Peak power"), "WRmax / Peak power"),
  build_meta_for_outcome(c("\\bTT\\b", "Time trial", "time trial", "time-trial"), "Time trial (TT)"),
  build_meta_for_outcome(c("MDA", "TBARS", "Thiobarbituric acid reactive substances"), "MDA +
TBARS"),
  build_meta_for_outcome(c("CK", "Creatine kinase"), "CK"),
  build_meta_for_outcome(c("AOPP", "advanced oxidation protein products"), "AOPP"),
  build_meta_for_outcome(c("LDH", "Lactate dehydrogenase"), "LDH")
)

rain_stats <- bind_rows(rain_list) %>%
  mutate(
    SE = (CI_ub - CI_lb) / (2 * 1.96)
  )

high_outcomes <- c("VO2max",
                   "TTE",
                   "WRmax / Peak power",
                   "Time trial (TT)",
```

```
                    "MDA + TBARS",
                    "CK")
low_outcomes  <- c("AOPP", "LDH")

rain_stats <- rain_stats %>%
  mutate(
    outcome = factor(
      outcome,
      levels = c(high_outcomes, low_outcomes)
    )
  ) %>%
  arrange(outcome) %>%
  mutate(
    y = as.numeric(outcome)
  )

set.seed(123)

rain_poly <- rain_stats %>%
  group_by(outcome, y, SMD, SE) %>%
  do({
    smd <- .$SMD
    se  <- .$SE
    yv  <- .$y

    x_seq <- seq(smd - 3 * se, smd + 3 * se, length.out = 200)
```

```
    dens  <- dnorm(x_seq, mean = smd, sd = se)

    dens_scaled <- dens / max(dens) * 0.35

    tibble(
      outcome = .$outcome,
      x       = x_seq,
      ymin    = yv - dens_scaled,
      ymax    = yv + dens_scaled
    )
  }) %>%
  ungroup()

fig_rain <- ggplot() +
  geom_ribbon(
    data = rain_poly,
    aes(x = x, ymin = ymin, ymax = ymax, group = outcome),
    fill  = "#4C72B0",
    alpha = 0.40
  ) +
  geom_errorbarh(
    data = rain_stats,
    aes(y = y, xmin = CI_lb, xmax = CI_ub),
    height    = 0.12,
    linewidth = 0.6,
    colour    = "grey40"
```

```
) +
geom_point(
  data = rain_stats,
  aes(x = SMD, y = y),
  shape  = 21,
  size   = 2.8,
  fill   = "#003366",
  colour = "white",
  stroke = 0.4
) +
geom_vline(xintercept = 0,
           linetype = "dashed",
           colour  = "grey60") +
scale_y_continuous(
  breaks = rain_stats$y,
  labels = rain_stats$outcome,
  expand = expansion(mult = c(0.05, 0.08))
) +
scale_x_continuous(
  name   = "Standardized mean difference (SMD)",
  expand = expansion(mult = c(0.08, 0.12))
) +
theme_nutrients() +
theme(
  axis.title.y = element_blank(),
  axis.text.y  = element_text(colour = "black"),
```

```
    plot.title   = element_text(
      face  = "bold",
      size  = 14,
      hjust = 0.5,
      colour = "#009966"
    ),
    plot.caption = element_text(
      hjust  = 0.5,
      size   = 8.5,
      margin = margin(t = 6)
    )
  ) +
  labs(
    title   = "Overall effects of algae supplementation on performance,\noxidative stress and muscle damage",
    caption = "Each raindrop represents the pooled SMD and its uncertainty for a given outcome.\nHigh-priority outcomes are listed on top; raindrop width reflects precision (narrower = more precise)."
  )

fig_rain
# ggsave("Fig5_Rainforest.png", fig_rain, width = 7, height = 6, dpi = 600)

## ---------------------------------------------------------
## Section J. Subgroup analyses (performance & muscle damage)
## ---------------------------------------------------------

run_subgroup_meta <- function(es_df, mod_var, panel_label) {
```

```
  df <- es_df %>%
    filter(!is.na(.data[[mod_var]]))
  if (nrow(df) == 0) return(NULL)

  sub_levels <- sort(unique(df[[mod_var]]))

  res_list <- lapply(sub_levels, function(lv) {
    df_lv <- df %>% filter(.data[[mod_var]] == lv)
    if (nrow(df_lv) == 0) return(NULL)

    fit <- rma(yi, vi, data = df_lv, method = "REML")

    tibble(
      panel      = panel_label,
      subgroup   = as.character(lv),
      SMD        = as.numeric(fit$b[1]),
      CI_lb      = fit$ci.lb,
      CI_ub      = fit$ci.ub,
      I2         = fit$I2,
      k          = fit$k
    )
  })

  bind_rows(res_list)
}
```

```
build_5mod_subgroups <- function(es_df) {
  sub_exercise <- run_subgroup_meta(es_df, "exercise_cat", "A. Exercise type")
  sub_algae    <- run_subgroup_meta(es_df, "algae_cat",    "B. Algae type")
  sub_dose     <- run_subgroup_meta(es_df, "dose_cat",     "C. Dose category")
  sub_duration <- run_subgroup_meta(es_df, "duration_cat", "D. Duration (weeks)")
  sub_form     <- run_subgroup_meta(es_df, "Fomulation",   "E. Formulation")


  sub_all <- bind_rows(sub_exercise,
                       sub_algae,
                       sub_dose,
                       sub_duration,
                       sub_form) %>%
    mutate(
      panel = factor(
        panel,
        levels = c("A. Exercise type",
                   "B. Algae type",
                   "C. Dose category",
                   "D. Duration (weeks)",
                   "E. Formulation")
      ),
      subgroup_label = sprintf("%s (k = %d)", subgroup, k)
    ) %>%
    group_by(panel) %>%
    mutate(
      subgroup_plot = fct_reorder(subgroup_label, SMD)
```

```
    ) %>%
    ungroup() %>%
    mutate(
      sig   = CI_lb > 0 | CI_ub < 0,
      label = sprintf("SMD = %.2f", SMD)
    )

  sub_all
}

plot_5mod_subgroups <- function(sub_all, main_title) {
  x_min   <- min(sub_all$CI_lb, na.rm = TRUE)
  x_max   <- max(sub_all$CI_ub, na.rm = TRUE)
  x_range <- x_max - x_min
  x_pad   <- 0.20 * x_range

  ggplot(sub_all,
         aes(x = SMD, y = subgroup_plot)) +
    geom_vline(xintercept = 0,
               linetype  = "dashed",
               colour    = "grey60") +
    geom_errorbarh(
      aes(xmin = CI_lb, xmax = CI_ub),
      height    = 0.15,
      linewidth = 0.5,
      colour    = "grey40"
```

```
) +
geom_point(
  shape  = 21,
  size   = 2.6,
  fill   = "#009966",
  colour = "black",
  stroke = 0.3
) +
geom_text(
  aes(x = CI_ub + 0.02 * x_range,
      label = label),
  hjust = 0,
  size  = 3
) +
scale_x_continuous(
  name   = "Standardized mean difference (SMD)",
  limits = c(x_min, x_max + x_pad)
) +
labs(title = main_title) +
facet_wrap(
  ~ panel,
  ncol   = 2,
  scales = "free_y"
) +
theme_nutrients() +
theme(
```

```r
    axis.title.y = element_blank(),
    axis.text.y  = element_text(colour = "black"),
    strip.text   = element_text(
      face   = "bold",
      size   = 10,
      colour = "white"
    )
  )
}

## Performance domain (VO2max, TTE, WRmax)
perf_patterns <- c(
  "VO2max", "VO₂max",
  "TTE", "Time to exhaustion", "time-to-exhaustion",
  "WRmax", "Maximal work rate", "Peak power"
)

dat_perf_sub <- dat %>%
  filter(
    str_detect(Indicator, str_c(perf_patterns, collapse = "|")),
    !is.na(`ΔMean_exp`), !is.na(`ΔSD_exp`),
    !is.na(`ΔMean_con`), !is.na(`ΔSD_con`)
  )

es_perf <- escalc(
  measure = "SMD",
```

```
  m1i = `ΔMean_exp`, sd1i = `ΔSD_exp`, n1i = n_exp,
  m2i = `ΔMean_con`, sd2i = `ΔSD_con`, n2i = n_con,
  data  = dat_perf_sub,
  vtype = "UB"
)

sub_perf_all <- build_5mod_subgroups(es_perf)

fig6_perf <- plot_5mod_subgroups(
  sub_perf_all,
  main_title = "Subgroup analyses of algae supplementation effects\non performance outcomes (VO2max, TTE, WRmax)"
)

fig6_perf
# ggsave("Fig6A_performance_subgroups.png", fig6_perf, width = 8, height = 6.5, dpi = 600)

## Muscle damage domain (CK + LDH)
damage_patterns <- c(
  "CK",  "Creatine kinase",
  "LDH", "Lactate dehydrogenase"
)

dat_damage_sub <- dat %>%
  filter(
    str_detect(Indicator, str_c(damage_patterns, collapse = "|")),
```

```
    !is.na(`ΔMean_exp`), !is.na(`ΔSD_exp`),
    !is.na(`ΔMean_con`), !is.na(`ΔSD_con`)
  )

es_damage <- escalc(
  measure = "SMD",
  m1i = `ΔMean_exp`, sd1i = `ΔSD_exp`, n1i = n_exp,
  m2i = `ΔMean_con`, sd2i = `ΔSD_con`, n2i = n_con,
  data  = dat_damage_sub,
  vtype = "UB"
)

sub_damage_all <- build_5mod_subgroups(es_damage)

fig6_damage <- plot_5mod_subgroups(
  sub_damage_all,
  main_title = "Subgroup analyses of algae supplementation effects\non muscle damage outcomes (CK,
LDH)"
)

fig6_damage
# ggsave("Fig6B_muscledamage_subgroups.png", fig6_damage, width = 8, height = 6.5, dpi = 600)

## ------------------------------------------------------
## Section K. Dose & duration meta-regression (performance)
## ------------------------------------------------------
```

```
mr_perf <- es_perf %>%
  filter(!is.na(dose_g_day),
         !is.na(duration_weeks)) %>%
  mutate(
    dose_mg_day   = dose_g_day * 1000,
    duration_days = duration_weeks * 7,
    weight        = 1 / vi,
    indicator_cat = case_when(
      str_detect(Indicator, regex("VO2", ignore_case = TRUE)) ~ "VO2max",
      str_detect(Indicator, regex("TTE", ignore_case = TRUE)) &
        !str_detect(Indicator, regex("TT", ignore_case = TRUE)) ~ "TTE",
      str_detect(Indicator, regex("TT", ignore_case = TRUE)) ~ "Time trial",
      str_detect(Indicator, regex("WR", ignore_case = TRUE)) |
        str_detect(Indicator, regex("Peak power", ignore_case = TRUE)) ~ "WRmax",
      TRUE ~ "Other"
    ),
    indicator_cat = factor(
      indicator_cat,
      levels = c("VO2max", "TTE", "WRmax", "Time trial", "Other")
    )
  )

## Meta-regression on dose (g/day)
fit_dose <- rma(
  yi, vi,
```

```
  mods   = ~ dose_g_day,
  data   = mr_perf,
  method = "REML"
)

new_dose <- seq(
  from = min(mr_perf$dose_g_day, na.rm = TRUE),
  to   = max(mr_perf$dose_g_day, na.rm = TRUE),
  length.out = 100
)

pred_dose <- predict(fit_dose, newmods = new_dose)

dose_line_df <- data.frame(
  dose_g_day = new_dose,
  pred       = pred_dose$pred,
  ci_lb      = pred_dose$ci.lb,
  ci_ub      = pred_dose$ci.ub
)

fig7A <- ggplot(mr_perf,
                aes(x = dose_g_day, y = yi)) +
  geom_ribbon(
    data  = dose_line_df,
    aes(x = dose_g_day, ymin = ci_lb, ymax = ci_ub),
    inherit.aes = FALSE,
```

```
  alpha = 0.15
) +
geom_line(
  data = dose_line_df,
  aes(x = dose_g_day, y = pred),
  inherit.aes = FALSE,
  linewidth = 0.7,
  colour = "#006633"
) +
geom_point(
  aes(size = sqrt(weight),
      fill = indicator_cat),
  shape  = 21,
  colour = "black",
  alpha  = 0.80,
  stroke = 0.25
) +
scale_size_continuous(name = "Study precision\n(√weight)",
                      range = c(2, 7),
                      guide = "legend") +
scale_fill_brewer(
  name   = "Outcome",
  palette = "Set2"
) +
labs(
  x     = "Dose of algae supplementation (g/day)",
```

```
    y     = "Standardized mean difference (SMD)",
    title = "Fig 7A. Meta-regression of performance outcomes\non algae dose"
  ) +
  theme_nutrients()

fig7A
# ggsave("Fig7A_meta_reg_dose.png", fig7A, width = 7, height = 5, dpi = 600)

## Meta-regression on duration (weeks)
fit_dur <- rma(
  yi, vi,
  mods   = ~ duration_weeks,
  data   = mr_perf,
  method = "REML"
)

new_dur <- seq(
  from = min(mr_perf$duration_weeks, na.rm = TRUE),
  to   = max(mr_perf$duration_weeks, na.rm = TRUE),
  length.out = 100
)

pred_dur <- predict(fit_dur, newmods = new_dur)

dur_line_df <- data.frame(
  duration_weeks = new_dur,
```

```
  pred          = pred_dur$pred,
  ci_lb         = pred_dur$ci.lb,
  ci_ub          = pred_dur$ci.ub
)

fig7B <- ggplot(mr_perf,
                aes(x = duration_weeks, y = yi)) +
  geom_ribbon(
    data  = dur_line_df,
    aes(x = duration_weeks, ymin = ci_lb, ymax = ci_ub),
    inherit.aes = FALSE,
    alpha = 0.15
  ) +
  geom_line(
    data = dur_line_df,
    aes(x = duration_weeks, y = pred),
    inherit.aes = FALSE,
    linewidth = 0.7,
    colour = "#006633"
  ) +
  geom_point(
    aes(size = sqrt(weight),
        fill = indicator_cat),
    shape  = 21,
    colour = "black",
    alpha  = 0.80,
```

```
    stroke = 0.25
  ) +
  scale_size_continuous(name = "Study precision\n(√weight)",
                        range = c(2, 7),
                        guide = "legend") +
  scale_fill_brewer(
    name    = "Outcome",
    palette = "Set2"
  ) +
  labs(
    x     = "Intervention duration (weeks)",
    y     = "Standardized mean difference (SMD)",
    title = "Fig 7B. Meta-regression of performance outcomes\non intervention duration"
  ) +
  theme_nutrients()


fig7B
# ggsave("Fig7B_meta_reg_duration.png", fig7B, width = 7, height = 5, dpi = 600)


## Dose–duration–effect bubble plot
fig7C <- ggplot(mr_perf,
                aes(x = dose_g_day,
                    y = duration_weeks)) +
  geom_point(
    aes(size = sqrt(weight),
        fill = yi),
```

```
  shape  = 21,
  colour = "black",
  alpha  = 0.85,
  stroke = 0.25
) +
scale_size_continuous(
  name  = "Study precision\n(√weight)",
  range = c(2, 7),
  guide = "legend"
) +
scale_fill_gradient2(
  name   = "SMD",
  low    = "#2166AC",
  mid    = "white",
  high   = "#B2182B",
  midpoint = 0
) +
labs(
  x     = "Dose of algae supplementation (g/day)",
  y     = "Intervention duration (weeks)",
  title = "Fig 7C. Dose–duration–effect bubble plot\nfor performance outcomes"
) +
theme_nutrients() +
theme(
  legend.box = "vertical"
)
```

```r
fig7C
# ggsave("Fig7C_dose_duration_bubble.png", fig7C, width = 7, height = 5.5, dpi = 600)


## ---------------------------------------------------------
## Section L. Radar plot + heatmap (Figure 9)
## ---------------------------------------------------------
## (Assumes 'radar_dat' and 'heatmap_dat' have been prepared beforehand.)


## Heatmap
max_abs <- max(abs(heatmap_dat$smd_benefit), na.rm = TRUE)


p_heat <- ggplot(heatmap_dat,
                 aes(x = indicator_group, y = Study, fill = smd_benefit)) +
  geom_tile(color = "white", linewidth = 0.3) +
  scale_fill_gradient2(
    low   = "#D73027",
    mid   = "#FEE090",
    high  = "#4575B4",
    midpoint = 0,
    limits   = c(-max_abs, max_abs),
    name = "SMD\n(benefit-oriented)"
  ) +
  labs(
    x = "Outcome indicator",
    y = "Study",
```

```
    title = "B. Effect-size heatmap"
  ) +
  theme_minimal(base_size = 11) +
  theme(
    panel.grid = element_blank(),
    axis.text.x = element_text(angle = 40, hjust = 1, vjust = 1),
    axis.text.y = element_text(size = 8),
    plot.title  = element_text(hjust = 0.5, face = "bold"),
    legend.position = "right"
  )

## Radar plot
radar_dat <- radar_dat %>%
  mutate(indicator_group = factor(indicator_group,
                                  levels = indicator_group))


radar_long <- radar_dat %>%
  select(indicator_group, smd_benefit)


radar_long_closed <- bind_rows(
  radar_long,
  radar_long[1, ]
)


p_radar <- ggplot(radar_long_closed,
                  aes(x = indicator_group, y = smd_benefit, group = 1)) +
```

```
  geom_polygon(fill = scales::alpha("#0072B2", 0.25),
               colour = "#0072B2", linewidth = 1.2) +
  geom_point(size = 2.3, colour = "#0072B2") +
  geom_hline(yintercept = 0, linetype = "dashed", colour = "grey70") +
  coord_polar() +
  ylim(0, max(radar_long$smd_benefit) * 1.15) +
  labs(
    x = NULL, y = NULL,
    title = "A. Radar plot of pooled beneficial effects"
  ) +
  theme_minimal(base_size = 11) +
  theme(
    panel.grid.major = element_line(colour = "grey85"),
    panel.grid.minor = element_blank(),
    axis.text.y = element_blank(),
    axis.text.x = element_text(size = 9),
    plot.title  = element_text(hjust = 0.5, face = "bold"),
    panel.background = element_rect(fill = "white", colour = NA)
  )

combined_fig9 <- p_radar + p_heat +
  plot_layout(widths = c(0.9, 1.1)) +
  plot_annotation(
    title = "Figure 9. Multidimensional visualization of seaweed/microalgae effects",
    theme = theme(
      plot.title = element_text(hjust = 0.5, face = "bold", size = 13)
```

```r
    )
  )

combined_fig9
# ggsave("Fig9_radar_heatmap.png", combined_fig9, width = 9, height = 5, dpi = 600)


## -------------------------------------------------------
## Section M. Supplementary Figures S5–S7 –
## CK leave-one-out, funnel plot and Egger test
## -------------------------------------------------------


theme_fig8 <- function(base_size = 10) {
  theme_minimal(base_size = base_size) +
    theme(
      panel.grid.major.y = element_blank(),
      panel.grid.minor   = element_blank(),
      panel.border       = element_rect(colour = "grey85", fill = NA, linewidth = 0.35),
      panel.grid.major.x = element_line(colour = "grey92", linewidth = 0.25),
      axis.title         = element_text(face = "bold", size = 9),
      axis.text          = element_text(colour = "black", size = 8),
      strip.background   = element_rect(fill = "#009966", colour = NA),
      strip.text         = element_text(colour = "white", face = "bold"),
      legend.position    = "bottom",
      plot.title         = element_text(size = 11, face = "bold")
    )
}
```

```
es_ck <- escalc(
  measure = "SMD",
  m1i = `ΔMean_exp`, sd1i = `ΔSD_exp`, n1i = n_exp,
  m2i = `ΔMean_con`, sd2i = `ΔSD_con`, n2i = n_con,
  data  = dat_ck,
  vtype = "UB"
)

fit_ck    <- rma(yi, vi, data = es_ck, method = "REML")
pooled_ck <- as.numeric(fit_ck$b[1])

### S5. Leave-one-out analysis (CK)

loo_ck <- leave1out(fit_ck)

loo_df <- data.frame(
  Study    = es_ck$Study,
  yi_omit = as.numeric(loo_ck$estimate),
  ci_lb    = loo_ck$ci.lb,
  ci_ub    = loo_ck$ci.ub
) %>%
  mutate(Study_plot = fct_reorder(Study, yi_omit))

x_lim_loo <- range(c(loo_df$ci_lb, loo_df$ci_ub), na.rm = TRUE)
x_lim_loo <- x_lim_loo + c(-0.05, 0.05)
```

```
p_loo_ck <- ggplot(loo_df,
                   aes(x = yi_omit, y = Study_plot)) +
  geom_vline(xintercept = pooled_ck,
             linetype   = "dashed",
             colour     = "grey75",
             linewidth  = 0.4) +
  geom_errorbarh(
    aes(xmin = ci_lb, xmax = ci_ub),
    height    = 0.12,
    linewidth = 0.4,
    colour    = "grey45"
  ) +
  geom_point(
    shape  = 21,
    size   = 2.0,
    fill   = "#009966",
    colour = "black",
    stroke = 0.25
  ) +
  scale_x_continuous(
    limits = x_lim_loo,
    expand = expansion(mult = c(0.05, 0.05))
  ) +
  labs(
    title = "Leave-one-out analysis of creatine kinase",
```

```
    x     = "Pooled SMD (leave-one-out)",
    y     = NULL
  ) +
  theme_fig8() +
  theme(
    axis.text.y = element_text(size = 8)
  )

p_loo_ck
# ggsave("Supp_Fig_S5_CK_LeaveOneOut.png", p_loo_ck, width = 4, height = 4, dpi = 600)

### S6. Funnel plot (CK)

funnel_df_ck <- as.data.frame(es_ck) %>%
  mutate(
    sei       = sqrt(vi),
    precision = 1 / sei
  )

prec_min <- min(funnel_df_ck$precision, na.rm = TRUE)
prec_max <- max(funnel_df_ck$precision, na.rm = TRUE)
prec_seq <- seq(prec_min, prec_max, length.out = 200)

z_975 <- qnorm(0.975)

tri_band_ck <- data.frame(
```

```
  precision = prec_seq,
  x_left    = pooled_ck - z_975 / prec_seq,
  x_right   = pooled_ck + z_975 / prec_seq
)

x_lim_fun <- range(
  c(tri_band_ck$x_left, tri_band_ck$x_right, funnel_df_ck$yi),
  na.rm = TRUE
)
x_pad     <- 0.06 * diff(x_lim_fun)
x_lim_fun <- x_lim_fun + c(-x_pad, x_pad)

egger_df_ck <- funnel_df_ck %>%
  mutate(SND = yi / sei)
egger_lm_ck <- lm(SND ~ precision, data = egger_df_ck)
egger_p_ck  <- signif(summary(egger_lm_ck)$coef[1, 4], 3)

p_funnel_ck <- ggplot() +
  geom_ribbon(
    data = tri_band_ck,
    aes(y = precision, xmin = x_left, xmax = x_right),
    fill   = "grey95",
    colour = NA
  ) +
  geom_line(
    data = tri_band_ck,
```

```
  aes(x = x_left, y = precision),
  colour   = "grey80",
  linewidth = 0.4
) +
geom_line(
  data = tri_band_ck,
  aes(x = x_right, y = precision),
  colour   = "grey80",
  linewidth = 0.4
) +
geom_vline(xintercept = pooled_ck,
           colour   = "grey40",
           linewidth = 0.6) +
geom_point(
  data   = funnel_df_ck,
  aes(x = yi, y = precision),
  shape  = 21,
  size   = 2.5,
  fill   = "#009966",
  colour = "black",
  stroke = 0.25
) +
scale_x_continuous(
  limits = x_lim_fun,
  expand = expansion(mult = c(0.02, 0.02)),
  name   = "Standardized mean difference (SMD)"
```

```
  ) +
  scale_y_continuous(
    name   = "Precision (1 / SE)",
    expand = expansion(mult = c(0.05, 0.08))
  ) +
  annotate(
    "text",
    x      = x_lim_fun[2] - 0.02 * diff(x_lim_fun),
    y      = prec_max - 0.04 * (prec_max - prec_min),
    label  = paste0("Egger p = ", egger_p_ck),
    hjust  = 1,
    vjust  = 1,
    size   = 3
  ) +
  labs(
    title = "Funnel plot for creatine kinase"
  ) +
  theme_fig8()

p_funnel_ck
# ggsave("Supp_Fig_S6_CK_Funnel.png", p_funnel_ck, width = 4, height = 4, dpi = 600)


### S7. Egger's regression test (CK)


prec_seq2_ck <- seq(
  from = min(egger_df_ck$precision, na.rm = TRUE),
```

```
  to   = max(egger_df_ck$precision, na.rm = TRUE),
  length.out = 100
)
pred_egger_ck <- data.frame(precision = prec_seq2_ck)
pred_egger_ck$SND_pred <- predict(egger_lm_ck, newdata = pred_egger_ck)

p_egger_ck <- ggplot(egger_df_ck,
                     aes(x = precision, y = SND)) +
  geom_hline(yintercept = 0,
             linetype  = "dashed",
             colour    = "grey40",
             linewidth = 0.6) +
  geom_point(
    shape  = 21,
    size   = 2.1,
    fill   = "#009966",
    colour = "black",
    stroke = 0.25
  ) +
  geom_line(
    data      = pred_egger_ck,
    aes(x = precision, y = SND_pred),
    colour    = "#006633",
    linewidth = 0.6
  ) +
  labs(
```

```
    title = "Egger's regression test for creatine kinase",
    x     = "Precision (1 / SE)",
    y     = "Standard normal deviate (SMD / SE)"
  ) +
  theme_fig8()


p_egger_ck
# ggsave("Supp_Fig_S7_CK_Egger.png", p_egger_ck, width = 4, height = 4, dpi = 600)
```

Section S10. PRISMA 2020 checklist

Section and Topic	Item #	Checklist item	Location where item is reported
TITLE			
Title	1	Identify the report as a systematic review.	Yes. Title page: "A Systematic Review and

Section and Topic	Item #	Checklist item	Location where item is reported
			Meta-Analysis".
**ABSTRACT**			
Abstract	2	See the PRISMA 2020 for Abstracts checklist.	**Yes.** Structured Abstract includes: Objective, Methods, Results, Conclusion.
**INTRODUCTION**			
Rationale	3	Describe the rationale for the review in the context of existing knowledge.	**Yes.** Introduction (paragraphs 1-3): Summarizes nutritional components, potential mechanisms, and highlights inconsistencies and gaps in existing literature.
Objectives	4	Provide an explicit statement of the objective(s) or question(s) the review addresses.	**Yes.** Final paragraph of Introduction: "This study aims to conduct a systematic review and meta-analysis..."
**METHODS**			
Eligibility criteria	5	Specify the inclusion and exclusion criteria for the review and how studies were grouped for the syntheses.	**Yes.** Section 2.3 "Inclusion and Exclusion Criteria (PICOS)" provides detailed criteria.
Information sources	6	Specify all databases, registers, websites, organisations, reference lists and other sources searched or consulted to identify studies. Specify the date when each source was last searched or consulted.	**Yes.** Section 2.2 lists PubMed, Web of Science, Embase, Cochrane, EBSCO, CNKI. Search date: June 2025.
Search strategy	7	Present the full search strategies for all databases, registers and websites, including any filters and limits used.	**Yes.** Section 2.2 describes strategy (keywords, Boolean operators). Complete strategies are in Supplementary Materials (Appendix 1).
Selection process	8	Specify the methods used to decide whether a study met the inclusion criteria of the review, including how many reviewers screened each record and each report retrieved, whether they worked independently, and if applicable, details of automation tools used in the process.	**Yes.** Section 2.2: Two researchers independent screening, conflicts resolved by a third. EndNote used for deduplication.
Data collection process	9	Specify the methods used to collect data from reports, including how many reviewers collected data from each report, whether they worked independently, any processes for obtaining or confirming data from study investigators, and if applicable, details of automation tools used in the process.	**Yes.** Section 2.4: Two independent reviewers extracted data using a pre-designed form, verified by a third. GetData Graph Digitizer used for graphical data.
Data items	10a	List and define all outcomes for which data were sought. Specify whether all results that were compatible with each outcome domain in each study were sought (e.g. for all measures, time points, analyses), and if not, the methods used to decide which results to collect.	**Yes.** Section 2.3 (Primary/Secondary outcomes) and 2.5 (prioritization of data points, e.g., 24-48h post-exercise).
	10b	List and define all other variables for which data were sought (e.g. participant and intervention characteristics, funding sources). Describe any assumptions made about any missing or unclear information.	**Yes.** Section 2.4 lists extracted data: author, year, sample, intervention details, outcomes, etc.
Study risk of bias assessment	11	Specify the methods used to assess risk of bias in the included studies, including details of the tool(s) used, how many reviewers assessed each study and whether they worked independently, and if applicable, details of automation tools used in the process.	**Yes.** Section 2.4: Cochrane RoB 2.0 tool used. Two independent reviewers, conflicts resolved by a third.
Effect measures	12	Specify for each outcome the effect measure(s) (e.g. risk ratio, mean difference) used in the synthesis or presentation of results.	**Yes.** Section 2.5: Standardized Mean Difference (SMD, Hedges' g) with 95% CI.
Synthesis methods	13a	Describe the processes used to decide which studies were eligible for each synthesis (e.g. tabulating the study intervention characteristics and comparing against the planned groups for each synthesis (item #5)).	**Yes.** Implied in Sections 2.3 & 2.5. Studies meeting PICOS criteria were synthesized by outcome (e.g., VO_2max, CK).
	13b	Describe any methods required to prepare the data for presentation or synthesis, such as handling of missing summary statistics, or data conversions.	**Yes.** Section 2.5: Conversion of SE/CI to SD, handling of crossover trials (r=0.5), combining multiple intervention arms.
	13c	Describe any methods used to tabulate or visually display results of individual studies and syntheses.	**Yes.** Section 2.5: R software (metafor package) used for forest plots, subgroup plots, etc.

Section and Topic	Item #	Checklist item	Location where item is reported
	13d	Describe any methods used to synthesize results and provide a rationale for the choice(s). If meta-analysis was performed, describe the model(s), method(s) to identify the presence and extent of statistical heterogeneity, and software package(s) used.	**Yes.** Section 2.5: Random-effects model due to expected clinical heterogeneity. Heterogeneity assessed via Cochran's Q and I^2 statistic (R software).
	13e	Describe any methods used to explore possible causes of heterogeneity among study results (e.g. subgroup analysis, meta-regression).	**Yes.** Section 2.6: Pre-specified subgroup analyses (exercise type, algae species, dose, etc.) and meta-regression
	13f	Describe any sensitivity analyses conducted to assess robustness of the synthesized results.	**Yes.** Section 2.6: Leave-one-out sensitivity analysis for primary outcomes
Reporting bias assessment	14	Describe any methods used to assess risk of bias due to missing results in a synthesis (arising from reporting biases).	**Yes.** Section 2.6: Funnel plots and Egger's test for outcomes with ≥10 studies.
Certainty assessment	15	Describe any methods used to assess certainty (or confidence) in the body of evidence for an outcome.	**Not formally assessed.** The confidence in the body of evidence was not rated using a framework like GRADE. Our assessment focused on risk of bias within studies and heterogeneity.
**RESULTS**			
Study selection	16a	Describe the results of the search and selection process, from the number of records identified in the search to the number of studies included in the review, ideally using a flow diagram.	**Yes.** Section 3.1 and **Figure 1** (PRISMA flow diagram).
	16b	Cite studies that might appear to meet the inclusion criteria, but which were excluded, and explain why they were excluded.	**Partially.** Figure 1 shows numbers excluded at full-text stage, but specific citations/reasons are not listed in main text.
Study characteristics	17	Cite each included study and present its characteristics.	**Yes.** Section 3.2 summarizes characteristics. **Table 1** provides detailed study-by-study data.
Risk of bias in studies	18	Present assessments of risk of bias for each included study.	Yes. Section 3.3 summarizes results. Figure 2 provides graphical summary (Risk of bias graph).
Results of individual studies	19	For all outcomes, present, for each study: (a) summary statistics for each group (where appropriate) and (b) an effect estimate and its precision (e.g. confidence/credible interval), ideally using structured tables or plots.	**Yes.** Forest plots (e.g., **Figures 3, 4**) show individual study SMDs and CIs. Table 1 includes key results.
Results of syntheses	20a	For each synthesis, briefly summarise the characteristics and risk of bias among contributing studies.	**Yes.** Narrative in Sections 3.4, 3.5. Subgroup analyses (Figure 6) show study groupings.
	20b	Present results of all statistical syntheses conducted. If meta-analysis was done, present for each the summary estimate and its precision (e.g. confidence/credible interval) and measures of statistical heterogeneity. If comparing groups, describe the direction of the effect.	**Yes.** Sections 3.4.1, 3.4.2, etc., report pooled SMD, 95% CI, and I^2 for each outcome (e.g., VO_2max SMD=0.88, I^2=80.8%). Figures 3-5 visualize these.
	20c	Present results of all investigations of possible causes of heterogeneity among study results.	Yes. Section 3.5 and Figures 6 & 7 present subgroup and meta-regression results.
	20d	Present results of all sensitivity analyses conducted to assess the robustness of the synthesized results.	**Yes.** Section 3.6 and **Figure 8A** (Leave-one-out analysis).
Reporting biases	21	Present assessments of risk of bias due to missing results (arising from reporting biases) for each synthesis assessed.	**Yes.** Section 3.6: Funnel plots (e.g., Figure 8B) and Egger's test (e.g., Figure 8C) indicate no significant bias for main outcomes.
Certainty of evidence	22	Present assessments of certainty (or confidence) in the body of evidence for each outcome assessed.	**Not formally assessed.** See explanation for Item 15. A systematic rating of the overall evidence quality for each outcome was not performed.
**DISCUSSION**			
Discussion	23a	Provide a general interpretation of the results in the context of other evidence.	**Yes.** Section 3.6: Funnel plots (e.g., Figure 8B) and Egger's test (e.g., Figure 8C) indicate no significant bias for main outcomes.

Section and Topic	Item #	Checklist item	Location where item is reported
	23b	Discuss any limitations of the evidence included in the review.	**Yes.** Section 4.5 (Study Limitations) discusses small sample sizes, heterogeneity, design variations, and baseline nutritional status.
	23c	Discuss any limitations of the review processes used.	**Yes.** Section 4.5 addresses limitations like language restriction (Chinese/English), reliance on certain biomarkers, and potential for publication bias.
	23d	Discuss implications of the results for practice, policy, and future research.	**Yes.** Throughout Discussion (4.2-4.4) and specifically in Section 4.5 (Future Directions) and Conclusion.
**OTHER INFORMATION**			
Registration and protocol	24a	Provide registration information for the review, including register name and registration number, or state that the review was not registered.	**Yes.** Section 2.1: Registered on PROSPERO (CRD420251166723).
	24b	Indicate where the review protocol can be accessed, or state that a protocol was not prepared.	**Yes.** Section 2.1 refers to the PROSPERO registration as the protocol.
	24c	Describe and explain any amendments to information provided at registration or in the protocol.	**Yes.** Section 2.1 describes minor adjustments (database combo, language, some subgroup analyses) made prior to data synthesis.
Support	25	Describe sources of financial or non-financial support for the review, and the role of the funders or sponsors in the review.	**Yes.** "Funding" section lists multiple grants. Role of funders: "The funders had no role in the design of the study...".
Competing interests	26	Declare any competing interests of review authors.	**Yes.** "Conflicts of Interest" section: "The authors declare no conflicts of interest."
Availability of data, code and other materials	27	Report which of the following are publicly available and where they can be found: template data collection forms; data extracted from included studies; data used for all analyses; analytic code; any other materials used in the review.	**Partially.** Supplementary Materials description mentions availability of R code (Appendix 9) and PRISMA checklist (Appendix 10). Data extraction forms/raw data availability is not explicitly stated.
